# Desalination Design Rules for Nanoporous 2D Materials: Low Edge Charges Sieve Ions, While Positive Edge Charges Exclude Boron

**DOI:** 10.1021/acsmaterialsau.6c00074

**Published:** 2026-08-31

**Authors:** Pradeep Dhondi, Sagar Ghorai, Ananth Govind Rajan

**Affiliations:** Department of Chemical Engineering, 29120 Indian Institute of Science, Bengaluru, Karnataka 560012, India

**Keywords:** nanoscale transport, 2D nanoporous materials, ion and boron rejection, molecular dynamics simulations, edge functionalization, membrane science

## Abstract

Molecular and ionic transport through nanopores in two-dimensional (2D) materials, which govern seawater desalination, are complex phenomena influenced by material chemistry, functional groups, and electrostatic interactions. We present a unified picture of nanoscale transport in 2D nanopores, demonstrating a pervasive principle valid across graphene, hexagonal boron nitride (hBN), and molybdenum disulfide (MoS_2_). That is, low edge charge effectively sieves ions, while positive edge charge excludes boron, which is regarded as the Achilles heel of seawater desalination. Quantum-mechanical calculations reveal that functional groups, such as hydrogen, hydroxyl, and oxo, modulate edge charges, tuning salt/boron rejection and water flux. Molecular simulations indicate that hydrogen-functionalized boron-terminated nanopores and oxygen-functionalized nitrogen-terminated hBN nanopores demonstrate superior salt rejection of close to 100%, due to roughly neutral edge atoms, which offer balanced ion rejection. This finding is found to persist with MoS_2_ and graphene nanopores, wherein low edge charge correlates with better salt rejection. Furthermore, hydrogen-functionalized nitrogen-terminated nanopores completely reject boron, a neutral contaminant that is difficult to remove from seawater, due to their positive edge charge. However, excessive positive charge compromises anion rejection and water permeance, despite repelling boric acid. These charge- and chemistry-mediated mechanisms provide a predictive basis and design rules for nanoporous 2D desalination membranes with high selectivity.

## Graphical abstract

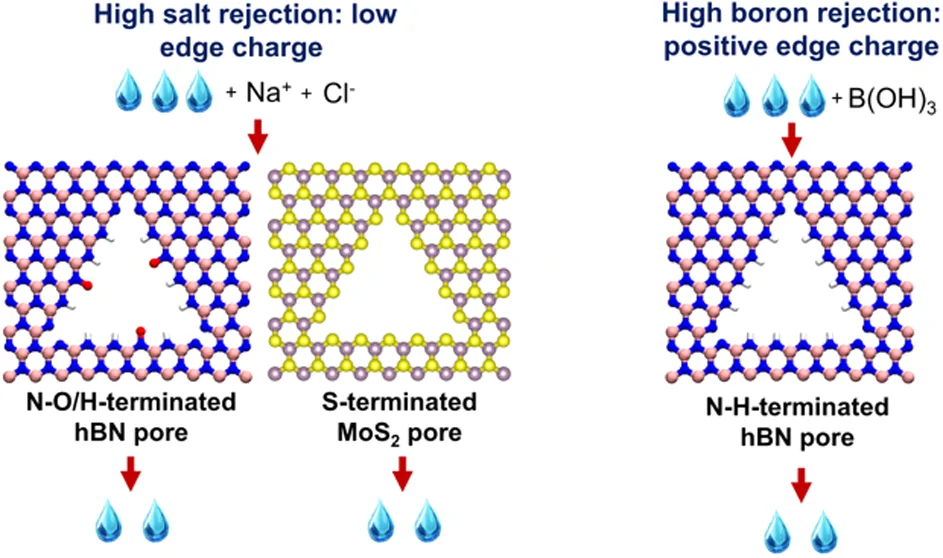

## Introduction

Two-dimensional (2D) materials have emerged as a class of advanced materials with exceptional properties, facilitating a wide range of applications.^1–4^ Among these, apart from graphene, nanoporous hexagonal boron nitride (hBN) and molybdenum disulfide (MoS_2_)^5–7^ have garnered significant attention due to their unique properties, such as high thermal stability, mechanical strength, and ion selectivity, attributed to their heterogeneous composition. Additionally, their atomically smooth surface and chemical inertness make them ideal candidates for separation processes, including seawater desalination and gas filtration, as well as for energy applications, such as osmotic energy harvesting.^8,9^ In this regard, the efficiency of 2D membranes is significantly influenced by their interactions with surrounding molecules, where surface characteristics, including defects, roughness, and functionalization, can have a profound impact on molecular transport.^10–12^ However, a clear understanding of these interfacial effects, which is crucial for improving process efficiencies and maximizing the potential of chemically modified 2D materials in various applications, remains under development.

Previously, researchers have explored the use of nanoporous graphene as a membrane for water desalination, aiming to remove salt and other impurities from seawater to make it suitable for drinking or industrial use.^13–22^ Others have also investigated its potential in applications such as gas separation and DNA detection.^23^ Recently, due to their aforementioned favorable properties, hBN and MoS_2_-based membranes have become an active area of research for desalination applications due to the materials’ high performance for both ion rejection and water permeance.^24,25^ Gao et al. used molecular dynamics (MD) simulations to investigate unfunctionalized triangular nanoporous hBN membranes for desalination.^26^ They found that pore size and edge termination significantly influence water flux and salt rejection, with nitrogen (N)-edged pores exhibiting higher permeance than boron (B)-edged pores, and medium-sized pores achieving 100% salt rejection with exceptional permeance.^26^ Additionally, they also showed that applying tensile strain to small pores enhanced water flux while maintaining ion rejection, demonstrating tunable desalination performance.^26^ Davoy et al. estimated that unfunctionalized nanoporous hBN exhibits higher water permeance than conventional polymer-based reverse osmosis (RO) membranes and even nanoporous graphene.^27^ Liu et al. used MD simulations to study water and ion transport through unfunctionalized hBN nanoslits. They found that water permeation follows classical hydrodynamics, while ion rejection depends on edge patterns. Armchair nanoslits showed minimal selectivity, whereas zigzag nanoslits blocked ions via electrostatic interactions. Crucially, partial charges on boron and nitrogen atoms significantly influenced water transport, affecting conduction rates.^28^ Later, Sharma et al. performed MD simulations and found that triangular-shaped nanopores with nitrogen-terminated edges in unfunctionalized, bicrystalline boron nitride exhibited higher water permeance with rejection close to 100%. They estimated the water permeance to be around 12–18 L cm^–2^ day^–1^ MPa^–1^, which is several times higher than RO membranes and graphene.^29^

In terms of work on MoS_2_, Heiranian et al. employed MD simulations^5^ to investigate desalination through unfunctionalized nanopores in single-layer MoS_2_ membranes. Their study showed that MoS_2_ nanopores can achieve extremely high water permeance, several orders of magnitude greater than conventional RO membranes, while maintaining significant salt rejection. They also demonstrated that the intrinsic pore edge structure and termination strongly influence transport behavior, enabling efficient water permeation through MoS_2_ nanopores. Cao et al. used MD simulations^7^ to examine water desalination through unfunctionalized nanopores in several 2D membranes, including MoS_2_, graphene, phosphorene, boron nitride, and MoSe_2_. They reported that single-layer MoS_2_ nanopores exhibit the highest water permeance while maintaining high ion rejection. The enhanced performance was attributed to the intrinsic atomic structure of the MoS_2_ pore edges, which promotes favorable water transport and lowers the energy barrier for permeation compared with other unfunctionalized 2D membranes. As can be seen, previous studies have focused on chemically unmodified hBN and MoS_2_, without any functional groups at nanopore edges, in stark contrast to the stable form of the material in aqueous media, which will inevitably contain various chemical groups, such as hydrogen, hydroxyl, and oxo.

Atomic-scale defects and surface roughness strongly influence the wetting and frictional properties of hBN and MoS_2_ surfaces, drawing significant attention in current research. Defects, specifically nanopores, play a crucial role in water desalination. These nanopores are necessary for water flow between the feed and permeate reservoirs, considering that the diameter of a water molecule is approximately 2.8 Å, which exceeds the available interstitial space in pristine 2D materials. Ryu et al. conducted experimental^31^ studies and demonstrated that subjecting an hBN monolayer to electron-beam irradiation led to the formation of B vacancies while maintaining the triangular shape of the pores, with their results supported by both MD and density functional theory (DFT). Additionally, Govind Rajan et al. utilized kinetic Monte Carlo simulations, DFT calculations, and graph theory^32^ to categorize the most prevalent nanopore shapes in hBN. Their findings also revealed that the most frequently observed and stable configurations were triangular-shaped nanopores. Other experimental studies have also demonstrated the feasibility of atomically precise nanopore formation in hBN.^33,34^ Nanopore formation has also been widely investigated in MoS_2_. Park et al. demonstrated^35^ the possibility of forming hexagonal nanopores in MoS_2_ using etching techniques combined with lithography. Furthermore, Bala et al. reported^36^ that defect creation in pristine MoS_2_ can lead to the formation of both hexagonal and triangular nanopores with either Mo- or S-terminated edges. In addition, Ryu et al. experimentally observed triangular oxidation-induced openings exposing both edge terminations, highlighting the different chemical reactivity of Mo and S edges.^37^ Thus, most studies have focused on investigating triangular and hexagonal nanopores in hBN and MoS_2_. However, despite the well-studied effects of different defects in hBN and MoS_2_ on various interfacial phenomena and desalination performance,^38–40^ the influence of hydrogen (H) and hydroxyl (OH) groups at hBN and MoS_2_ edges remains poorly understood, as studies considered water and ion permeation via unfunctionalized, i.e., bare nanoporous hBN and MoS_2_,^5,7,26,27,29,41,42^ although such a configuration is experimentally unrealistic.

Our previous ab initio molecular dynamics (AIMD) simulations^25,43^ demonstrated that bare 2D nanopores are inherently unstable in aqueous environments, highlighting the critical need for accurate modeling of functionalized nanopores for desalination applications. More importantly, the investigation of functionalized hBN and MoS_2_ interfaces holds significant promise due to the distinctive electrostatic-field modifications these functional groups introduce.^44–46^ In this work, to bridge the knowledge gaps regarding the role of functional groups in modulating water, ion, and molecular transport through 2D nanopores, we benchmarked the performance of functionalized nanopores for desalination applications using DFT-supported MD simulations. By considering realistic models of nanopores, we show that the nanopore edge charge crucially determines ion/molecular rejection. We also examine the generalization of the proposed principle across 2D materials, such as hBN, MoS_2_, and graphene, including both functionalized and bare pores. As outlined by Cohen-Tanugi and Grossman, charge serves as a tunable and complementary parameter that modulates the local electrostatic field and hydration environment, particularly when steric exclusion alone is insufficient.^47^ However, we advance here a new, unifying principle that *high edge charge compromises counterion rejection, while low edge charge promotes balanced ion rejection*, leading to highly selective membranes. In this regard, we demonstrate that hydrogen atoms at boron edges and oxygen groups at nitrogen edges (with low edge charge) can lead to both high water flux and high ion rejection. Moreover, we show that *highly positively charged edge atoms repel boric acid molecules*, which are emerging contaminants in seawater. Notably, boron has been identified as a difficult-to-reject neutral species that can have detrimental effects on the health of plants, animals, and humans.^48,49^ Overall, our work demonstrates that it is possible to exploit differences in electronegativity (or lack thereof) between edge atoms and functional group atoms to obtain high (or low) edge charges, which can enable high boron and ion rejection, through nanoporous 2D membranes.

## Results and Discussion

### Superiority of Low-Edge-Charge Nanopores for Simultaneously High Salt Rejection and Water Permeation

MD simulations were performed on hydrogen-functionalized and unfunctionalized hBN nanopores terminated by boron and nitrogen atoms. The simulation setup depicting a nanoporous hBN membrane separating the feed and the permeate sides is shown in Figure 1a. The various configurations employed in the simulations, distinguishing functionalized and unfunctionalized nanopores of various 2D materials, are shown in Figure 1b.

**Figure 1:**
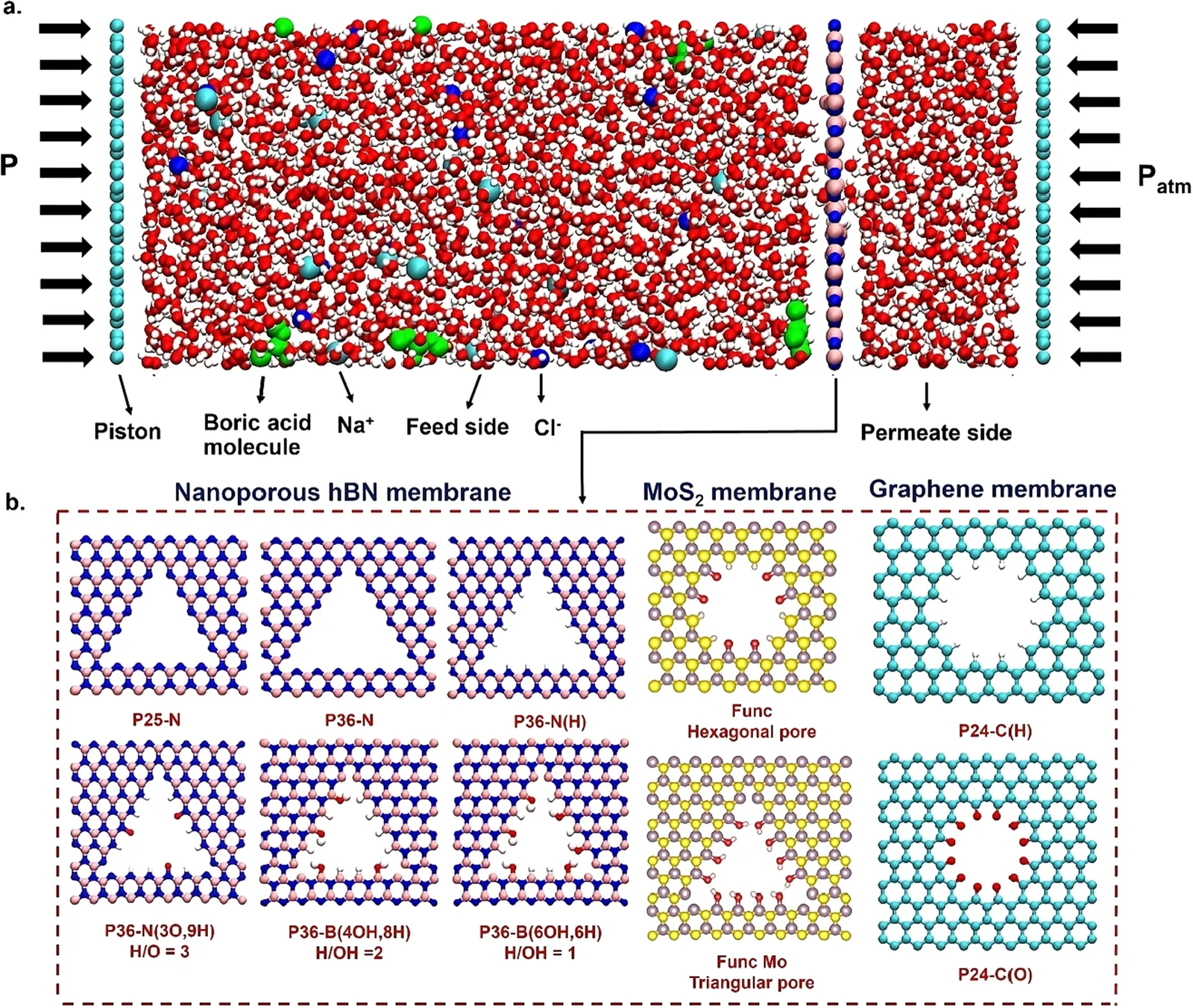
Overview of the nanopores investigated in this work for seawater desalination. (a) Schematic of the water desalination simulation setup, where a nanoporous hBN membrane separates the feed and permeate sides, enabling boron and NaCl salt separation under an applied pressure of 50 MPa, with the system confined by graphene pistons. (b) Different nanoporous 2D materials used in the present study: hBN: P25–N (pore with {15 B, 10 N} atoms removed exposing N edges), P36–N (pore with {21 B, 15 N} atoms removed exposing N edges), P36–N­(H) (P36 functionalized with 12 hydrogen atoms on the edge N atoms), P36–N­(3O,9H) (P36 with 25% oxygen and 75% hydrogen functionalization at the N edges), P36–B­(4OH,8H), and P36–B­(6OH,6H); MoS_2_: hexagonal pore with 36 {24 S, 12 Mo} atoms removed, functionalized with 12 H and 6 O groups on the S and Mo edge atoms, respectively and triangular Mo-terminated pore with 57 {45 S, 12 Mo} atoms removed, functionalized with 12 OH groups on the Mo edge atoms; graphene: P24–C­(H) with 24 carbon atoms removed, functionalized with 12 hydrogen atoms and P24–C­(O) with 24 carbon atoms removed, functionalized with 12 oxygen atoms, i.e., quinone groups. Color code: B: pink, N: blue, H: white, O: red, C: cyan, Mo: purple, S: yellow.

In the rest of the paper, we use the notation P*n-X* for unfunctionalized hBN pores formed by removing *n* atoms, giving rise to *X* (B or N) terminated edges. For example, P16–N designates a triangular nanopore with 10 B and 6 N atoms removed, giving rise to an N-terminated pore. For functionalized nanopores, the nomenclature used is P*n*-*X*(*mY*), where *n* denotes the total number of atoms removed, and *m* denotes the number of functional groups of type *Y*. For example, a P16–B pore with H functionalization is referred to as P16–B­(6H). Note that edge atoms, which are part of five-membered rings, do not functionalize as shown in Figure 1b.^43^ Thus, in a P16–B pore with 12 rim atoms, only 6 of them can harbor functional groups. In other words, a P16–B­(12H) pore is not possible, but a P16–B­(6H) pore exists where all sterically accessible sites are functionalized. In such cases, when all sterically accessible sites are functionalized, we omit the number *m* for brevity. Thus, a P16–B­(6H) pore is referred to simply as a P16–B­(H) pore. Moving forward, in cases involving more than one functional group (e.g., *Y*_1_ and *Y*_2_), the format is P*n*-*X*(*m*_1_ *Y*_1_,*m*_2_ *Y*_2_), where *m*_1_ and *m*_2_ represent the number of *Y*_1_ and *Y*_2_, respectively. For example, P36–N­(3O,9H) corresponds to a N-terminated triangular nanopore with 36 atoms removed, featuring 3 O and 9 H functional groups at the pore edges.

As inferred from Figure 2a,c and Section S1 of the Supporting Information, the water permeance follows the trend P36-*N* > P36–B > P25–*N* > P25–B > P36–B­(H) > P16–N ≈ P36–N­(H) > P25–N­(H) > P25–B­(H) > P16–N­(H) for a NaCl solution. This indicates that the highest water flux occurs through unfunctionalized pores (created by removing 36 and 25 atoms from hBN), with larger nanopores facilitating faster water transport. Notably, the triangular pore with 36 removed atoms has not been previously explored, as prior studies have primarily examined pores with up to 25 removed atoms.^26,27,29,41^ Our recent work focused solely on hydrogen-functionalized P36 pores,^43^ whereas this study also examines other functional groups and the P25/P16 pores to assess their effectiveness in optimizing salt rejection and water flux. Interestingly, unfunctionalized N-terminated pores exhibit slightly higher water permeance than their B-terminated counterparts. However, despite earlier studies considering unfunctionalized hBN, our previous AIMD simulations revealed that such structures are unstable in an aqueous environment.^43^ Therefore, evaluating the effect of nanopore functionalization on water transport and salt rejection is crucial. As shown in Figure 2a,c, hydrogen functionalization reduces water flow by a factor of approximately 3 to 5.

**Figure 2:**
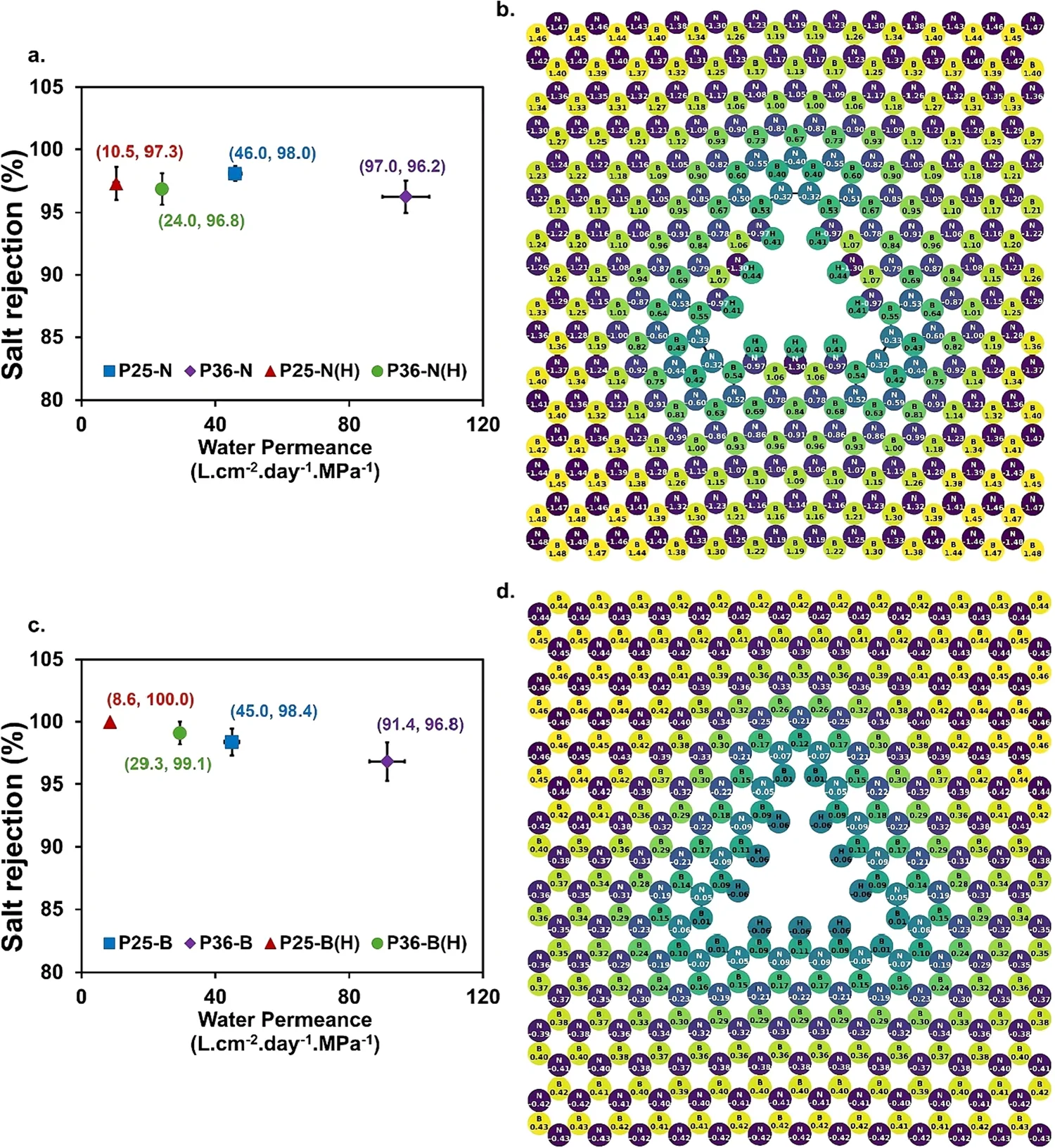
MD simulation results for the separation of NaCl aqueous salt solution through nanopores of varying sizes and edge terminations (N and B) with H functionalization. (a, c) Water permeance per unit membrane area (L cm^–2^ day^–1^ MPa^–1^) from the feed side to the permeate side and the overall salt rejection percentage for N-terminated (a) and B-terminated (c) nanopores. (b, d) Partial charge distribution (in elementary charge units, e) for the P25–N­(H) pore (b) and the P25–B­(H) pore (d). Details regarding how the partial charges are obtained are provided in the Methods section. In all hBN models, the nanopore density was ∼0.088 nm^–2^.

To understand the water permeance trend through different nanopores, we calculated the one-dimensional (1D) potential of mean force (PMF) using equilibrium water density profiles. In Section S2, we present a detailed analysis of the PMF and the calculated water transport barrier across the H-functionalized pores, which qualitatively explains the trend observed for the water flux through the various nanopores considered. Later in the paper, we also perform hydrogen bond analysis for interfacial water near the pores to provide a deeper understanding of the observed water permeance trends across the different pores, offering additional insights into the factors governing water transport.

In addition to the water flux, salt rejection is a crucial membrane parameter that is extremely important to design high-efficiency desalination processes.^50^ For real-world desalination, membranes must reach ion rejection levels exceeding 99.5%, making near-perfect salt exclusion a higher priority than simply enhancing water permeability.^50^ Out of the N-terminated pores, the highest salt rejection rates of 99.1 and 100% are for P16–N and P16–N­(H) pores, respectively (Figure 2a and Section S1). However, the former pore cannot be observed in reality (due to the lack of functionalization)^43^ and the latter pore has a very low water permeance of 2.7 L cm^–2^ MPa^–1^ day^–1^ (Section S1). The low water permeance of P16–N­(H) is due to its smaller pore diameter of approximately 4.15 Å, as compared to the diameter of a water molecule, which is about 2.8 Å. The average pore limiting diameters, obtained from five independent simulations for each pore considered in this study, are reported in Section S3, calculated using image analysis of nanopores rendered with each atom represented as a circle with a radius equal to its van der Waals radius. For every simulation, the pore limiting diameter was evaluated at each trajectory frame and subsequently time-averaged to get a single characteristic value for that run. The values presented in Section S3 represent the mean of these time-averaged diameters across five independent simulations. We found that the functionalization of hBN nanopores changes the rejection percentage of individual ions through the membrane. For instance, throughout the simulations, only one Na^+^ ion permeated through the small P16–N pore, that too likely due to the negative charge on the terminal N atoms. This changed to no permeated Na^+^ ions for the P16–N­(H) pore, due to the positive hydrogen atoms at the edges repelling the cations, as well as the pore’s smaller size. With an increase in nanopore size, the negatively charged Cl^–^ ions also permeated with one Cl^–^ ion and a few Na^+^ ions passing through the P25–N pore in all six simulations conducted with different initial configurations, resulting in 98% salt rejection. The individual ion rejections (Na^+^, Cl^–^) are given in Section S4. Thus, as the pore size increases (P16–N to P36–N), both Na^+^ and Cl^–^ ions pass through the membrane, with more Na^+^ ions passing, resulting in a decrease in the total ion rejection for larger pore sizes, as can be observed from Figure 2a.

Considering a P25–N pore, the negatively charged N atoms (−0.41e to −0.45e and −0.13e to −0.14e for the six corner atoms) at the rim of an unfunctionalized pore allow more number of Na^+^ ions to permeate than Cl^–^ and the reverse happens with hydrogen functionalization (P25–N­(H) pore) as the H atoms bear positive charges (0.41e to 0.44e), as seen in Figure 2b and Section S5. Despite functionalization reducing the effective pore size, having a similar magnitude and a significant amount of charge on the pore edge could be the reason we observe nearly identical poor performance in terms of ion rejection between bare and H-functionalized N-terminated pores, with the former compromising Na^+^ rejection and the latter Cl^–^ rejection. A similar trend is observed for larger pores, where the charge distribution of the H-functionalized and unfunctionalized P36 pores for N- and B-terminated configurations is shown in Section S5. For the larger unfunctionalized pores (P36 and P25), the calculated properties are averaged over six different initial configurations of the desalination system.

For the B-terminated pores, the water permeance follows the same order as that of the N-edge pores, i.e., P36–B > P25–B> P36–B­(H) > P25–B­(H) for a NaCl salt solution (Figure 2c). The charges on the bare edge boron atoms vary between −0.28e to −0.48e and −0.07e to 0.01e for the six corner atoms (Section S5) near the pore, whereas the charges on the H-functionalized B atoms are −0.06e (Figure 2d). We observed slightly higher salt rejection rates with B-terminated unfunctionalized nanopores for the cases with nanopores P36 and P25 in the hBN membrane. This can be rationalized by the lower magnitude of partial charges on the B edge atoms as compared to charges on the N atoms at N edges. Surprisingly, a significant difference was observed in the case of H-functionalized B edges, where the salt rejection is nearly 100% due to the very small magnitude of charge on H atoms at the pore edges (−0.06e), with a higher water permeance compared to hydrogen-terminated N edge pores. This is because, when terminal pore atoms are charged, they preferably allow the counterions to pass through, compromising salt rejection. On the other hand, uncharged terminal atoms are not particularly favorable for anions or cations to pass through, and thus improve the overall salt rejection!

In desalination studies, achieving a membrane design that balances both rejection and water permeance is crucial. In this regard, the P36–B­(H) pore offers a water flux of 29.3 L cm^–2^ MPa^–1^ day^–1^ with a rejection of 99.1%, whereas the P25–B­(H) pore exhibits a water flux of 8.6 L cm^–2^ MPa^–1^ day^–1^ with a 100% salt rejection. This corresponds to an order of magnitude and 4-fold increase in water flux, respectively, as compared to the H-functionalized N-terminated pore with the highest salt rejection (P16–N­(H) pore offering 100% salt rejection and a water flux of 2.7 L cm^–2^ MPa^–1^ day^–1^). Note that the water permeance with current RO membranes is below 0.1 L cm^–2^ MPa^–1^ day^–1^,^51–57^ and with reasonable 2D nanopore densities about an order of magnitude lower than those simulated here, the water flux through nanoporous hBN would still be higher. This finding implies that B-terminated H-functionalized pores can be ideal for water desalination as they enable both high water permeance and salt rejection, due to roughly neutral H atoms at their edges.

### Insights Into the Impact of Edge Charge on Pore-Ion Interactions

To further understand the superior ion rejection of H-functionalized B-terminated pores, we evaluated the Lennard-Jones (LJ) and Coulombic (Coul) energy contributions for Na^+^ and Cl^–^ by decomposing the time-averaged total potential energy (TPE) into interactions with both the full hBN sheet and the functionalized pore edges (Section S6). For surface-level interactions, we saw that Coulombic interactions dominate. In P36–N­(H), a Na^+^ ion encounters a mild repulsive barrier (15 kcal/mol), and the Cl^–^ ion is weakly stabilized (−18 kcal/mol), while in P36–B­(H), the trend reverses: Na^+^ is attracted (−10 kcal/mol) and Cl^–^ is slightly repelled (+10 kcal/mol). While the overall interactions with the hBN sheet govern ion accumulation near the membrane, ion translocation is primarily controlled by the discrete charge distribution at the pore edge. In this regard, in P36–N­(H), where hydrogen (+0.41e to +0.44e) is bonded to negatively charged nitrogen, Na^+^ experiences a steep repulsive barrier from the edge charges (+179 kcal/mol), while Cl^–^ is stabilized in a deep well by the same charges (−183 kcal/mol), promoting selective Cl^–^ passage. In P36–B­(H), with a weakly negative edge charge (−0.06e), Na^+^ is moderately stabilized by the edge charges (−30.5 kcal/mol) and Cl^–^ is repelled (+31 kcal/mol); yet both ions remain blocked, Cl^–^ by repulsion, Na^+^ by energetic trapping. The LJ contributions remain minor (−0.08 to −0.6 kcal/mol) in all cases, confirming that Coulombic interactions dominate the ion-pore energetics, whence the magnitude of edge charge plays a critical role in modulating transport rates.

### Tuning Membrane Performance through Oxygen-Containing Functional Groups

Next, we investigated the water desalination performance of functionalized nanopores by incorporating oxygen-containing functional groups at the P36­(B/N) pore edges. Cohen-Tanugi and Grossman^47^ showed that functionalized graphene with polar groups like OH at nanopore edges offers high salt rejection and water permeance, surpassing the performance of current RO and nanofiltration membranes.^15,58–60^ However, the potential of hBN for similar functionalization (OH at B edges and O at N edges) has not been explored due to the lack of models for such functional groups in hBN prior to the present work. Notably, even in its unfunctionalized form, hBN nanopores exhibit superior water permeance and salt rejection compared to unfunctionalized graphene and MoS_2_.^5,15,29^ In our prior study, detailed analyses using AIMD simulations of N and B-terminated nanopores in aqueous environments demonstrated the possibility of 2 N–O and 10 N–H groups for the P36–N pore, and 6 B–H and 6 B–OH groups for the P36–B pore.^43^ Therefore, we checked the salt rejection performance with different numbers of N–O/N–H and B–OH/B–H groups for P36–N and P36–B nanopores, respectively. To examine the effect of increasing the number of oxygen-containing functional groups, we considered four P36–N pores with increasing O content (Figure 3a,b). We found that the P36–N­(2O,10H) and P36–N­(3O,9H) pores exhibit higher salt rejection and water flux due to the low magnitude of charge on H (0.04–0.1e) and O (−0.12e to −0.13e) atoms, as seen in Figure 3a,b, and Section S5. For instance, as shown in Figure 3a, P36–N­(3O,9H) exhibited high water flux (despite the pore diameter being only around 4.85 Å, see Section S3) with a water permeance of 29.6 L cm^–2^ MPa^–1^ day^–1^ and 100% salt rejection.

**Figure 3:**
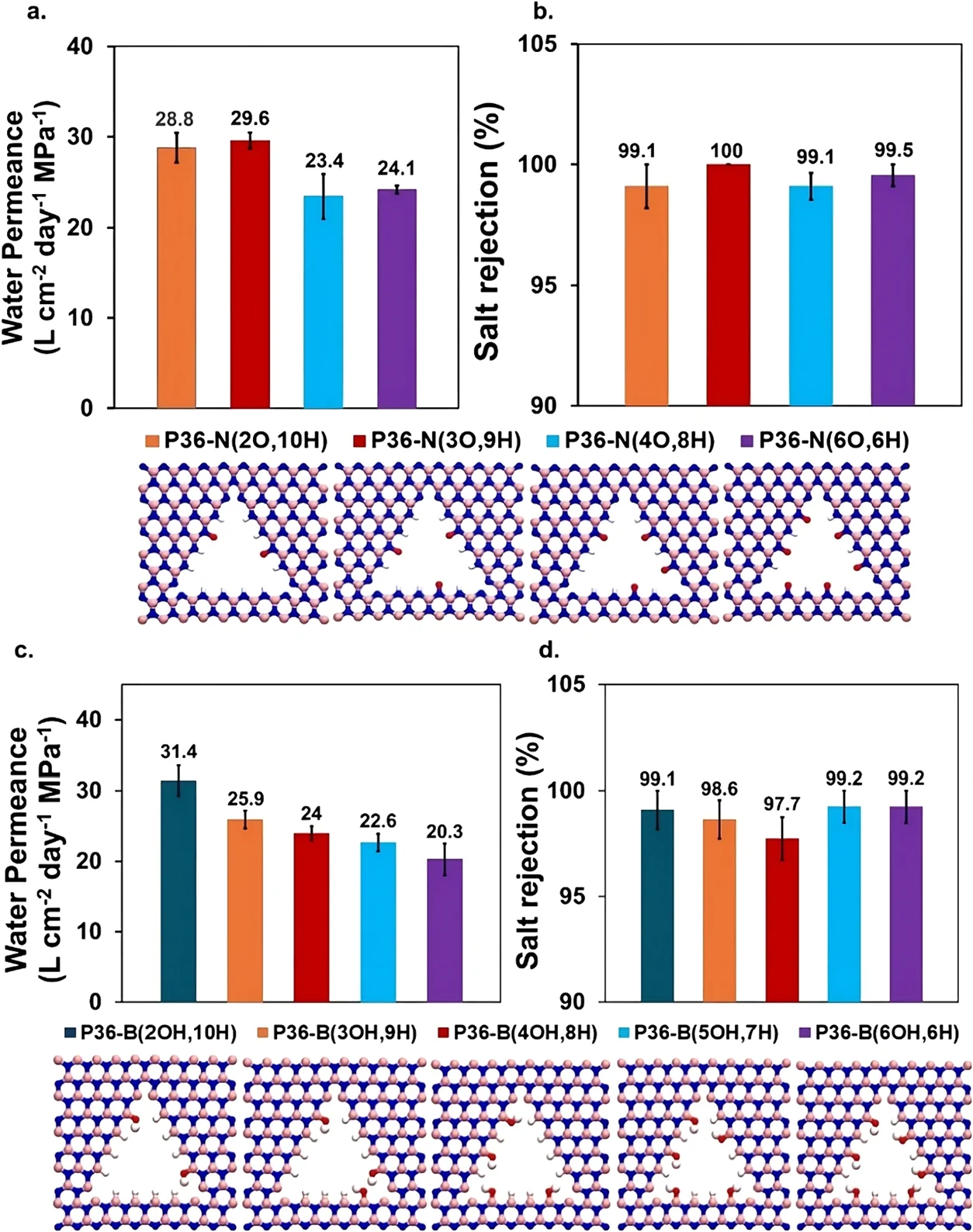
MD simulation results for NaCl aqueous salt solution separation for various nanopore sizes and edge terminations with OH/O/H functionalization. (a, c) Water permeance per unit membrane area (L cm^–2^ day^–1^ MPa^–1^) from the feed side to the permeate side for N-terminated (a) and B-terminated (c) nanopores. (b, d) Overall salt rejection percentage for N-terminated (b) and B-terminated (d) nanopores. In all the considered hBN models, the nanopore concentration (i.e., pore density) in the hBN layer was ∼0.088 nm^–2^. Color code: B: pink, N: blue, H: white, O: red.

Thus, similar to the B–H groups, the introduction of N–O groups can allow an increase in both water flux and ion rejection. This optimal performance is due to the low edge charge near the nanopore, which decreases the hydrogen bond lifetime of water, leading to more efficient water transport, as discussed in the subsequent section. Similarly, in the case of P36–B pores, we considered five configurations (Figure 3c,d). Although our AIMD simulations^43^ suggest equal (6:6) H and OH functionalization, the functionalization/pore creation procedure employed (e.g., hydrogen/oxygen plasma or ozone etching) may result in more H or more OH functionalization. Therefore, we varied the number of OH groups from 2 to 6. The functionalized pore structures employed in the MD simulations were selected from the lowest-energy DFT configurations and are consistent with the stable edge-functionalized structures observed in our previous AIMD simulations.^43^ As an example, different configurations of the P36–B­(6OH,6H) pore and their relative stabilities are provided in Section S7 of the Supporting Information.

We found that the P36–B­(6OH,6H) pore exhibits the highest rejection of 99.2 ± 0.7% among OH/H-functionalized B-terminated pores (Figure 3d), due to a smaller magnitude of charge on the edge atoms: O atoms with −0.13e to −0.31e charge, H atoms in the OH group with 0.02e to 0.19e charge, and the H atoms attached to boron atoms (HB) with −0.04e to −0.09e charge (Section S5), but it showed decreased water permeance (Figure 3c) due to the reduced pore size with increasing OH percentage (Section S3). The individual ion rejections (Na^+^, Cl^–^) through pores with oxygen-containing functional groups at the edges are given in Section S4. Overall, we conclude that pores with N–O groups are particularly suited for water desalination applications, similar to pores with B–H functional groups, as revealed in the previous subsection.

### Hydrogen Bond Dynamics to Rationalize Water Transport via Functionalized B- and N-Terminated Nanopores

As discussed above, in seawater desalination membranes, functional groups at the edges of nanopores, such as hydroxyl (−OH), H, and oxygen attached to nitrogen (−ON) atoms, can play a crucial role in water transport and salt rejection. These groups modify the chemical environment at the nanopore, influencing water permeation by forming hydrogen bonds with water molecules while permeating through nanopores. The strength and lifetime of these hydrogen bonds directly affect the efficiency of water filtration, as they control how water molecules interact with the membrane and each other. Hydrogen bond lifetime analysis is essential for understanding how long a water molecule remains influenced by a functionalized site before diffusing away.^61^ A longer hydrogen bond lifetime at the nanopore can slow down water transport. Conversely, shorter-lived hydrogen bonds promote rapid water permeation, increasing the flux. To quantify water transport across functionalized nanopores, we used MDAnalysis^62,63^ to compute hydrogen bond lifetimes via the time autocorrelation function, $C\left(\tau \right)=\left\langle \frac{h_{\mathrm{ij}}\left(t_{0}\right)h_{\mathrm{ij}}\left(t_{0}+\tau \right)}{h_{\mathrm{ij}}{\left(t_{0}\right)}^{2}}\right\rangle$ , where angular brackets represent time averaging with different time origins *t*_0_, *h_ij_* (*t*) = 1 if a hydrogen bond exists between atoms *i* and *j* at time *t*, and *h*_*ij*_ = 0 otherwise. The decay of *C*(τ) over time reflects bond stability, with a slower decay indicating persistent hydrogen bonds and a faster decay suggesting dynamic, transient interactions. Multiple time origins *t*_0_ are used for statistical accuracy. To obtain the hydrogen bond lifetime (τ), we fit a single-exponential function to the autocorrelation curve as $C\left(t\right)=A\exp\left(\frac{-t}{\tau }\right)$ , where *A* is the maximum amplitude of the autocorrelation and τ is the hydrogen bond lifetime. The characteristic time constant τ provides insight into how long a water molecule remains hydrogen-bonded before breaking and reforming elsewhere.

In Figure 4, we illustrate the hydrogen bond analysis (HBA) for various functionalized nanopores, highlighting their role in water transport. In all the HBA calculations, a maximum value of 3.5 Å for the donor oxygen-acceptor oxygen distance and a maximum value of 30° (i.e., minimum value of 150°) for the donor oxygen–hydrogen-acceptor oxygen angle were used to screen for hydrogen bonds.^64–66^ The P36–B­(H) and P36–N­(3O,9H) pores, with hydrogen bond lifetimes of 0.87 and 0.91 ps, respectively, enable higher water flux (Figures 2c and 3a). In contrast, the P36–B­(6OH,6H) and P36–N­(H) pores exhibit lower permeance due to longer hydrogen bond lifetimes of 1.42 and 1.15 ps, respectively, which hinder water flow (Figures 2a and 3c). This trend aligns with the number of hydrogen bonds formed at functionalized edges during the simulation. As expected, the B–H pore forms minimal hydrogen bonds, as boron’s low electronegativity (with resulting low partial charges between 0.09 and 0.12e) makes it a weak hydrogen bond acceptor.^67^ However, the P36–N­(H) pore forms more hydrogen bonds due to the high electronegativity of the nitrogen atom (which carries a charge of −1.45e), leading to stronger water interactions and further reducing permeance. For the P36–B­(6OH,6H) pore, the smaller diameter also contributes to reduced water transport, along with the effect of hydrogen bonding (Section S3). This highlights the critical role of functional groups at the nanopore in regulating water transport, where their interactions govern both permeance and selectivity, shaping overall filtration efficiency. Note that the hydrogen-bond analysis is performed in a nonequilibrium simulation to understand its relationship with water transport. We start from the final configuration of each equilibrium simulation, where the water molecules are well organized, as reflected by the high number of hydrogen bonds at zero time in the insets of Figure 4. After the 50 MPa feed-side pressure is applied, the system adjusts to nonequilibrium conditions, leading to a decrease in the hydrogen-bond count before reaching a stable mean value.

**Figure 4:**
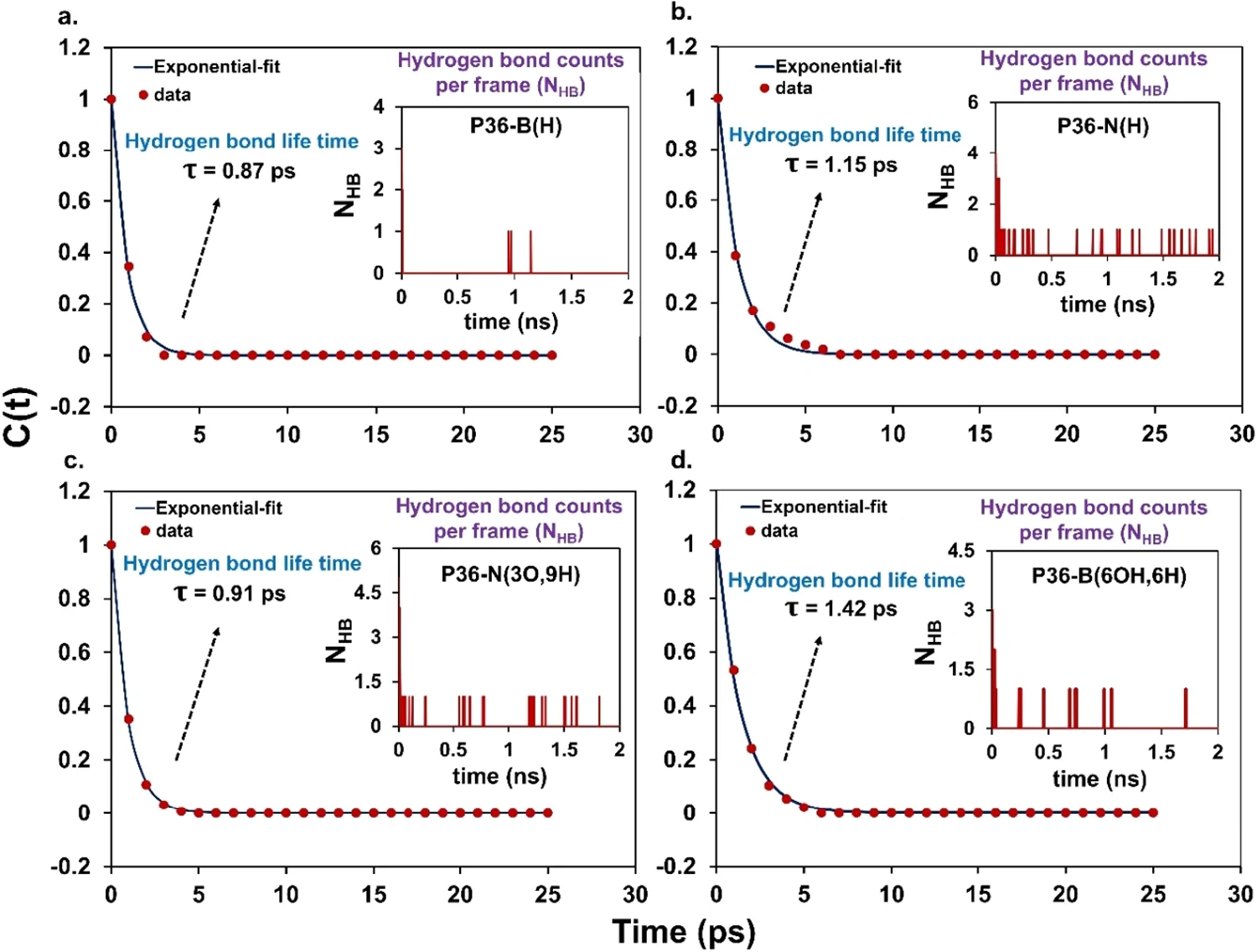
Hydrogen bond lifetime analysis of various functionalized nanopores, showing the correlation between hydrogen bond lifetime and hydrogen bond count with water permeance in MD simulations. (a) P36–B­(H) pore with a hydrogen bond lifetime of 0.87 ps. (b) P36–N­(H) pore with a hydrogen bond lifetime of 1.15 ps. (c) P36–N­(3O,9H) pore with a hydrogen bond lifetime of 0.91 ps. (d) P36–B­(6OH,6H) pore with a hydrogen bond lifetime of 1.42 ps. *N*_HB_ is the number of hydrogen bonds formed during the simulation time.

### Superiority of Positive-Edge-Charge Nanopores for Boron Rejection

We next carried out MD simulations to understand boron rejection by H, OH, and O functionalized pores with N and B edges. Note that boron is present in the form of boric acid (B­(OH)_3_) in seawater,^68^ and is a serious contaminant that poses severe health risks to humans and animals.^69–71^ Only at very high pH, boric acid forms the borate ion (B­(OH)_4_^–^). Thus, boron exists in uncharged form in seawater, whose pH is around 8.1, making it very challenging to remove.^68,72–74^ Our results, shown in Figure 5, clearly demonstrate that nanoporous hBN with hydrogen-functionalized nitrogen-terminated edges can act as a boron-rejecting membrane. The P36–N­(H) pore achieves high water flux (Figure 5a) along with 100% boric acid rejection (Figure 5b), with no boric acid molecules passing through to the permeate side even after 5 ns, as shown in Figure 5c.

**Figure 5:**
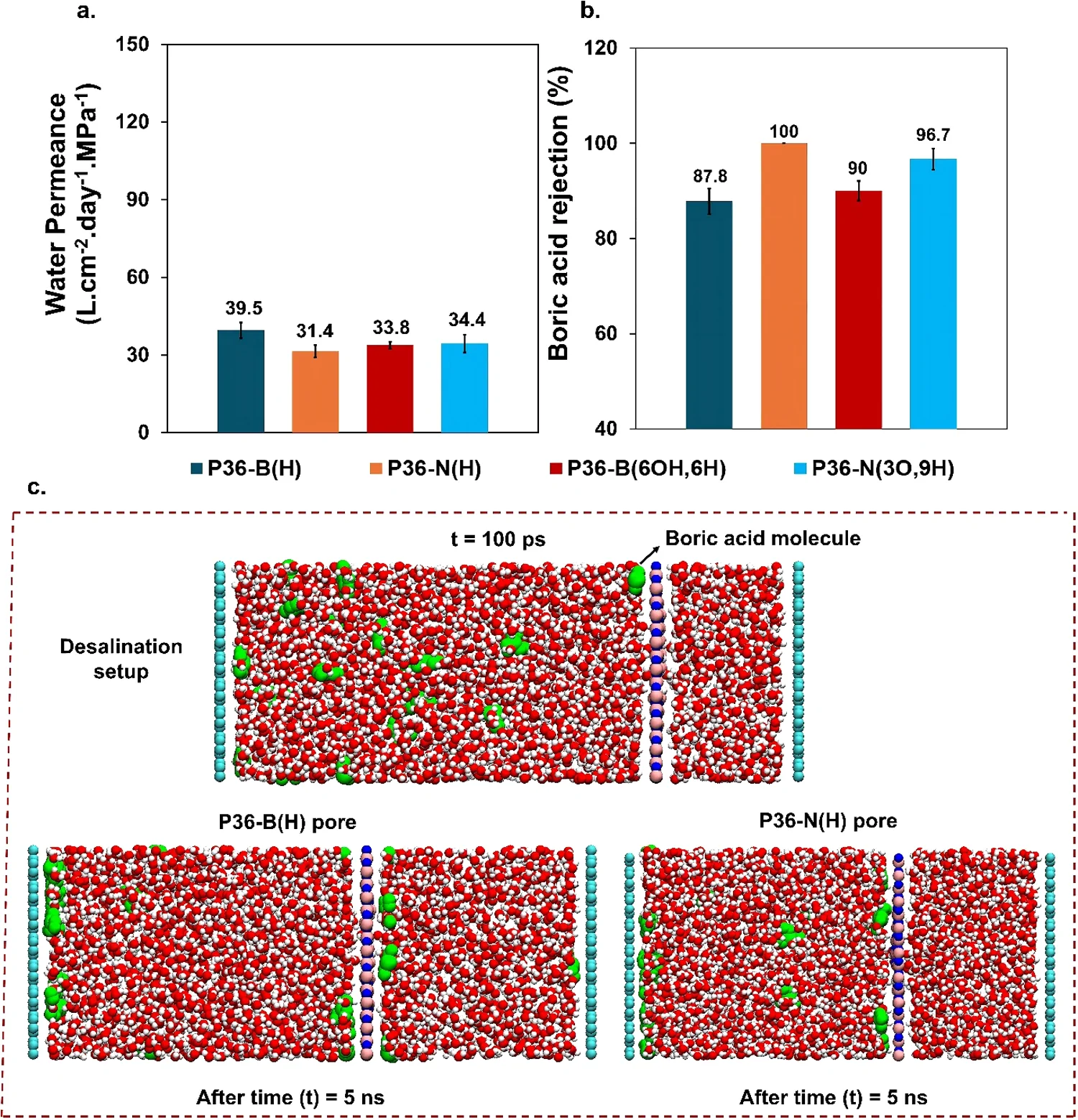
MD simulation results for boric acid separation for P36 nanopores with B/N edges and OH/O/H functionalization. (a) Water permeance per unit membrane area (L cm^–2^ day^–1^ MPa^–1^) from the feed side to the permeate side for functionalized N-terminated and B-terminated nanopores. (b) Boric acid rejection percentage for functionalized N-terminated and B-terminated nanopores. (c) MD simulation snapshots of P36–N­(H) and P36–B­(H) pores before and after a 5 ns run. The initial configuration contains 18 boric acid molecules (green) in the feed side, and after 5 ns of simulation time, five boric acid molecules pass through the P36–B­(H) nanopore, while none traverse the P36–N­(H) pore. In all the considered hBN models, the nanopore concentration (i.e., pore density) in the hBN layer was ∼0.088 nm^–2^. Note that transient accumulation of boric acid molecules near graphene and hBN was observed in all pore systems at certain times during the simulations. However, these molecules do not remain permanently adsorbed and continuously exchange with the bulk solution, indicating a dynamic equilibrium between boric acid molecules near the surfaces and those dissolved in the bulk phase.

This superior performance is due to the positively charged hydrogen atoms at the nitrogen edges, which create strong electrostatic interactions with H atoms in boric acid (having a charge of 0.21e) that prevent the molecules from passing through the membrane. On the other hand, when the pore edges are negatively charged (as in P36–B­(H) and P36–B­(6OH,6H)), the trend is reversed. This behavior is illustrated in Figure 5a,b, where P36–N­(H) and P36–N­(3O,9H) exhibit greater boric acid rejection due to the presence of positively charged atoms at the pore edges. This finding again reinforces the crucial role played by edge charge in determining the performance of seawater desalination membranes.

### Exploring Trends Across Various 2D Materials

Recently, Risplendi et al. explored graphene as a membrane for boric acid separation.^75^ Their research showed that an appreciable water flux of 8.4 L cm^–2^ day^–1^ MPa^–1^ with a pore size of 8.37 Å was accompanied by a low boron rejection rate of 80%. When they reduced the nanopore size to 3.60 Å, they were able to obtain a boron rejection rate of 100% for boron, but the water flux dropped to zero! Although the study by Risplendi et al. considered only positively charged graphene pores (hydrogen-functionalized carbon-terminated), we performed MD simulations with both charge types (C–O terminated and C–H terminated) and observed the same trend as hBN: *a positively charged pore* (P24–C­(H)) *blocks more boron than a negatively charged one* (P24–C­(O)), but the boron rejections are nowhere as close as hBN and peak around ∼89%, compared to 100% for hBN. Representative AIMD snapshots provided in Section S8 suggest that both C=O and C–H edge terminations may coexist in functionalized graphene nanopores under aqueous conditions. Accordingly, the fully O-functionalized and fully H-functionalized graphene pores considered in the present work represent two limiting cases designed to systematically isolate the influence of edge chemistry on transport behavior, while mixed edge functionalization can be investigated in future studies. Notably, the charges on the edge atoms are higher in graphene than in B-terminated hBN, indicating the superiority of the latter pores in rejecting ions. Note that in the MD simulations, the pore size was kept similar to that of the hBN membrane to enable direct comparison of performance between the two materials. Results for functionalized graphene and pore diameters for different graphene models considered are presented in Section S8. It is evident from our study that nanoporous N-terminated and H-functionalized hBN achieves high water permeance and 100% boric acid rejection (with pore P36–N­(H)) with a high water flux of 31.4 L cm^–2^ day^–1^ MPa^–1^ and can thus act as an efficient boron-rejecting membrane, highlighting its superior performance compared to graphene.

Recognizing the importance of the edge charge in modulating ion and molecular transport in functionalized and bare 2D materials, we next analyzed correlations between ion/boron rejection and edge charge. In Figure 6, we show that the influence of edge charge on salt and boric acid rejection is a general behavior across different 2D materials such as hBN, graphene, and MoS_2_. A higher magnitude of edge charge generally leads to lower salt rejection (Figure 6a), while negatively charged nanopores result in reduced boric acid rejection (Figure 6b). Note that the average edge charge reported in Figure 6 solely considers the outermost set of atoms (e.g., only H in OH functionalization) as these primarily determine electrostatic fields. For Figure 6a, the horizontal axis corresponds to the average absolute nanopore edge charge, i.e., $\frac{1}{N}\sum_{i}^{N}\left|q_{i}\right|$ , while for Figure 6b (and Figure 7), it corresponds to the average nanopore edge charge (i.e., $\frac{1}{N}\sum_{i}^{N}q_{i}$ ), where *i* runs over the *N* outermost nanopore edge atoms considered in the calculation.

**Figure 6:**
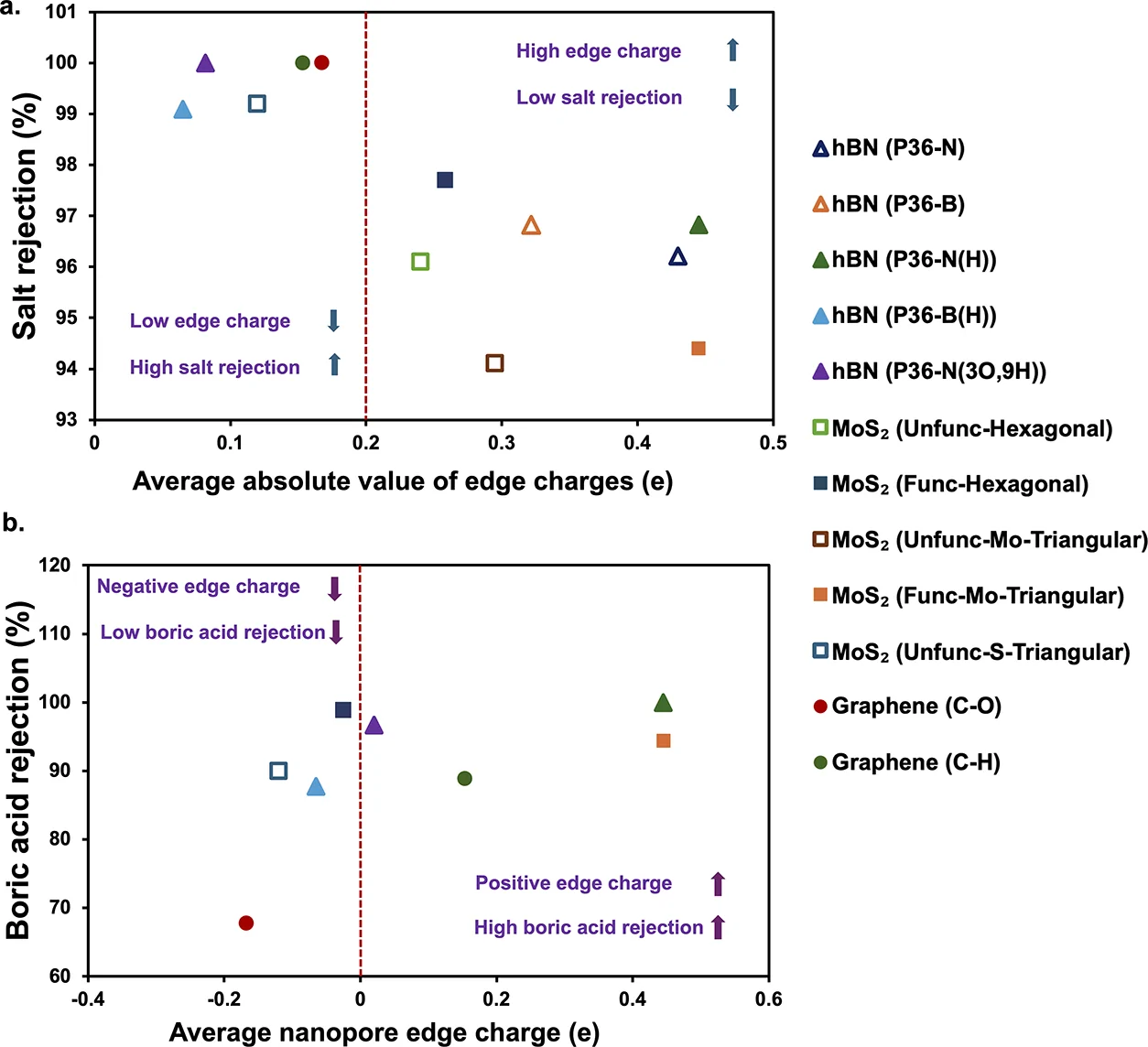
Influence of edge charge sign and magnitude on boric acid and salt rejection. (a) Effect of the average absolute nanopore edge charge on the salt rejection percentage for hBN, MoS_2_, and graphene nanoporous 2D membranes. (b) Effect of the average nanopore edge charge on boric acid rejection for hBN, MoS_2_, and graphene nanoporous 2D membranes. Data points are represented by different marker shapes: triangles for hBN, squares for MoS_2_, and circles for graphene. Filled markers denote functionalized pores, whereas unfilled markers indicate unfunctionalized pores. MD results for MoS_2_ are taken from Dhondi et al.^25^

Interestingly, if one plots the water permeance per unit pore area (not membrane area) versus the average value of the edge charges, one sees that negative charges lead to more water permeation (Figure 7), which is exactly analogous to the case of more boron passing at negative edge charges (Figure 6b)! Indeed, both water and boric acid have terminal H atoms, which implies that the proposed unifying principle regarding positive edge charge repelling molecules with positive terminal atoms holds across molecules. This finding implies that, when corrected for the flow area, the water flux crucially depends on the sign and magnitude of edge charges on the 2D nanopores. For example, although the negatively charged P36–B­(H) pore possesses a smaller pore limiting diameter (∼5.8 Å) than the positively charged P36–N­(H) pore (∼6.4 Å), it still exhibits higher water permeation and ion rejection, suggesting that pore-edge electrostatics may play an important role along with local steric effects and functional group chemistry in governing nanopore transport. To confirm these trends, we examined the relationship between the average edge charge and water permeance, boric acid rejection, and salt rejection using Pearson’s correlation analysis. A coefficient of +1 indicates a perfect positive correlation, while −1 denotes a perfect negative correlation. From Figures 6 and 7, we observe a strong negative correlation between the average absolute value of edge charges and salt rejection (−0.782), and a negative correlation between the average nanopore edge charge and water permeance (−0.580), indicating that higher edge charge leads to reduced ion rejection and water transport. In contrast, a positive correlation (+0.555) is observed between boric acid rejection and the average nanopore edge charge, suggesting that enhanced edge charges favor selective boric acid rejection.

**Figure 7:**
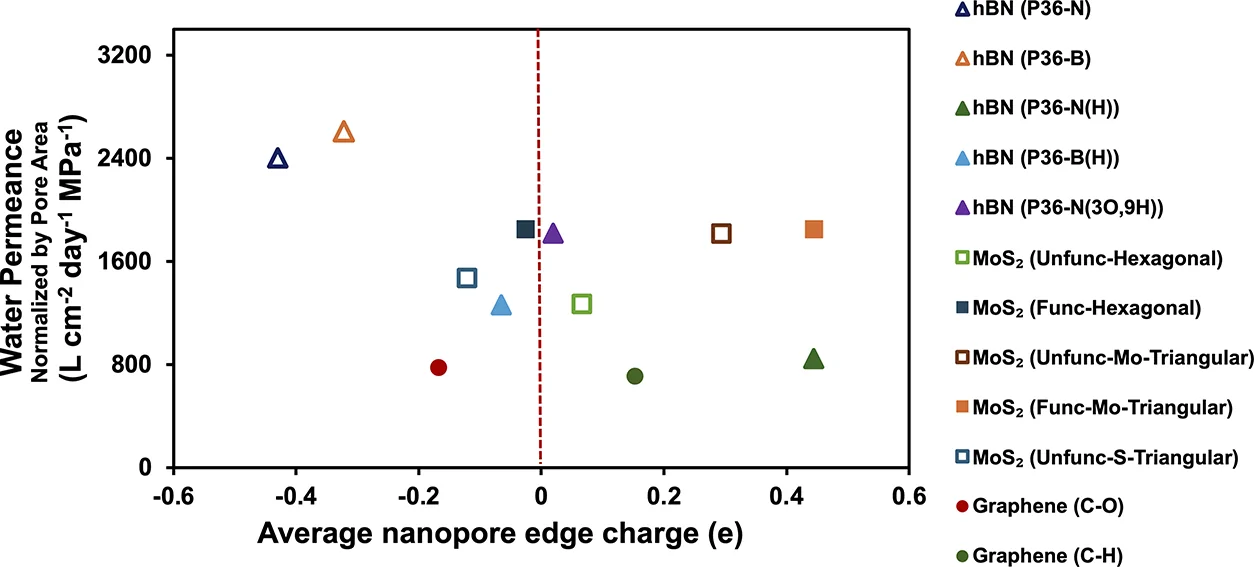
Water permeance per unit average pore limiting area (L cm^–2^ day^–1^ MPa^–1^) versus the average value of the edge charges, showing reduced water flux with positive edge charges. MD results for MoS_2_ are taken from Dhondi et al.^25^

To further understand the trends in water permeance and solute rejection (in NaCl and boric acid solutions) via functionalized hBN nanopores, we examined the hydration structure of the solutes using the radial distribution function (RDF), g­(*r*). As shown in Section S9, water–water interactions are slightly stronger in the boric acid solution (higher g­(*r*) peak), indicating more structured water due to the presence of boric acid molecules. This is further supported by the RDF plots therein, where NaCl shows a higher first hydration-shell peak, indicating stronger ion–water interactions than boric acid–water interactions. As a result, water transport is slightly reduced for NaCl. In contrast, boric acid shows weaker hydration, leading to higher water permeance. However, boric acid’s neutral charge and weak hydration shell also make it more likely to pass through the nanopore, compared to ions. Additional RDF analysis details are provided in Section S9. Note that elevated boric acid concentrations were employed to obtain statistically meaningful boric acid-pore interaction events within accessible MD simulation times, consistent with prior MD studies of boron transport through nanoporous membranes.^75^ Simulations performed at lower boric acid concentration for the P36–B­(H) and P36–N­(H) pores also showed similar overall trends, as discussed in Section S10. Overall, functionalized hBN nanopores offer high water flux and ion/boron rejection, making them strong candidates to surpass current RO desalination membranes. Note, however, that, while classical force fields have provided consistent and physically meaningful results in this study, future work could explore machine learning potentials^76–78^ finetuned for functionalized 2D nanopores in water to further improve accuracy.

## Conclusions

In summary, this work addresses the critical role of edge charge modulation by chemical functionalization in heteropolar 2D materials, such as hBN and MoS_2_, as well as in homopolar graphene, in achieving excellent seawater desalination performance. Using accurate ab initio force fields, quantum mechanically derived atomic charges, and detailed molecular dynamics simulations, we demonstrated that the edge charge sign and magnitude plays a crucial role in determining the water flux, salt rejection, and boron exclusion across nanopore edge chemistries. We observed that *pores with lower edge charge exhibit the highest salt rejection* because they do not favor the permeation of any particular ions (anions or cations) due to electrostatics. In this regard, for the case of the hBN system, B-terminated H-functionalized nanopores were found to outperform N-terminated H-functionalized pores. We also examined the effect of introducing oxygen-containing functional groups. We found that the presence of N–O groups is particularly favorable, competing with the B–H terminated nanopores for the top position in terms of membrane performance due to low edge charge on H and O atoms. Similarly, for the MoS_2_ system, we observed that unfunctionalized S-terminated nanopores exhibit high water permeance and strong salt rejection. This behavior arises from the relatively low and negative edge charge of the sulfur atoms, which facilitates water transport while inhibiting ion permeation.^25^

Additionally, through hydrogen bond lifetime analysis and PMF calculations, we rationalized how water permeance varies with different functional groups at the nanoporous edges. Furthermore, our study also showed that nanoporous 2D materials with *positive edge charge can function well as boron-rejecting membranes*. For instance, in hBN, the hydrogen-functionalized N-terminated pore perfectly rejected boric acid (100% rejection) with enhanced water permeance. In contrast, graphene and MoS_2_ nanopores demonstrated boron rejection efficiencies of 89 and 98%, respectively, while reduced water flux was observed for the graphene nanopore. We explained this finding by the strong electrostatic interactions between positive charges at nanopore edges and the hydrogen in the OH group of boric acid. On the other hand, the hydrogen-functionalized B-terminated pore enabled high water permeance and 100% salt rejection but low boric acid rejection due to negatively charged terminal hydrogen atoms. We also showed that the principle of positive edge charge leading to lower molecular flux holds not only for boric acid, but also for water, which also has positive terminal atoms. These findings highlight the impact of edge atom charges, as modulated by functional group moieties, on molecular/ion selectivity in heteropolar and homopolar 2D materials. Going forward, the pH of the solution can be an important tunable parameter that could be used to favor one type of functional group versus another (e.g., H at acidic pH vs OH under alkaline pH), and this aspect could be explored. Overall, our work demonstrates that a careful synthesis of functionalized nanoporous 2D materials can provide a route to develop high ion-rejection or high boron-rejection membranes. In conclusion, our comprehensive investigation of nanoporous 2D materials elucidates the influence of functional groups on seawater desalination performance and highlights the critical role of edge atom charges in governing water, ion, and boron transport through nanoporous membranes. We hope that our work motivates the tuning of functional groups to develop highly efficient 2D desalination membranes that enable high salt and boron rejection, as well as rapid water permeance.

## Computational Methods

### Computational Framework for DFT and Charge Calculations

Electronic density functional theory (DFT) calculations were performed using the CP2K package (version 7.1) with the mixed Gaussian and plane wave (GPW) method.^79^ The Perdew–Burke–Ernzerhof (PBE) functional,^80^ within the generalized gradient approximation (GGA), was used to describe exchange-correlation interactions. Molecularly optimized (MOLOPT) double-ζ basis sets^81^ were used for valence electrons, and Goedecker-Teter-Hutter (GTH) pseudopotentials were used for the nuclei and core electrons.^82^ Dispersion corrections were included using Grimme’s D3 method.^83^ A plane-wave cutoff energy (E_CUT) of 450 Ry and a relative cutoff energy (REL_CUT) of 60 Ry were employed for the GPW calculations. For the supercells studied, a Γ-point (1 × 1 × 1) *k*-point mesh was used for Brillouin-zone sampling. Partial charges on atoms were calculated using the density-derived atomic point (DDAP) charges approach, with the default DDAP parameters in cp2k for the cutoff in reciprocal space (GCUT), number of Gaussians (NUM_GAUSS), progression factor for Gaussian exponents (PFACTOR), and the minimum radius of the Gaussians employed (MIN_RADIUS).^84^ We validated the obtained charges by making sure that the quantum-mechanical electrostatic potential felt by a test charge passing through the center of the pore was faithfully reproduced by the point charges. In all cases, rescaling of charges was required to match the DFT-calculated and point-charge-based electrostatic potentials. Detailed information on the computational methods and charge calculations can be found in Section S11, wherein the rescaling factors used to generate the rescaled charges for various pores are also tabulated. We ensured that the resulting charges are physical, i.e., B atoms are positive and N/O atoms are negative, although there could be minor charge reversal for some atoms. For the P36–B­(6OH,6H) pore, we tested a different DDAP parameter set (Case 1) to ensure that the charges far away from the pore reach close to the bulk B/N charges in hBN (≈ ± 0.91e). Note that it was not possible to achieve this for all the pores. The charge distribution for the P36–B­(6OH,6H) pore, using the “Case 1” DDAP parameters, is depicted in Section S12, along with the hydrogen bond lifetime for water near the pore. The corresponding results and DDAP parameters are also presented therein. We found that the water flux and salt rejection increased slightly, remaining within the error bars, implying that different charge distributions that match the electrostatic potential give rise to similar water flux and salt rejection. Hence, we went ahead with the default DDAP parameters for all the pores investigated in this work.

### MD Simulations

MD simulations were performed using the Large-scale Atomic/Molecular Massively Parallel Simulator (LAMMPS) package^85,86^ (version dated 29 Sep 2021) to provide insights into the water desalination performance. The force fields employed included harmonic bonds and angles, harmonic impropers, CHARMM-style dihedrals, 6–12 Lennard-Jones (LJ) parameters, and partial charges. The force field parameters for boron (B) and nitrogen (N) atoms in hBN, including both bonded and nonbonded interactions, were adopted from Govind Rajan et al.^87^ Note that our prior study has validated the force field for bulk hBN by calculating the water slip length (∼1 nm) and contact angle (∼66°) in the presence of surface roughness, demonstrating remarkable agreement with experimental observations.^40^ Bonded and nonbonded parameters for functional groups (OH, O, and H) at the pore edges were obtained from our previous work.^43^ Parameters for sodium (Na^+^) and chloride (Cl^–^) ions were adapted from Zeron et al.,^88^ and carbon atoms in the pistons were modeled using LJ parameters from Cheng and Steele.^89^ Water molecules were represented by the TIP4P/2005 four-site model,^90^ and interaction parameters for boric acid molecules were taken from the data provided by Risplendi et al.^75^ Interactions between different types of atoms were calculated using geometric mean combining rules, i.e., $\epsilon_{\mathrm{ij}}=\sqrt{\epsilon_{\mathrm{ii}}\epsilon_{\mathrm{jj}}}$ and $\sigma_{\mathrm{ij}}=\sqrt{\sigma_{\mathrm{ii}}\sigma_{\mathrm{jj}}}$ , where ϵ denotes the depth of the LJ potential well and σ represents the distance at which the LJ potential is zero. A complete list of the force-field parameters employed is provided in Section S13.

The initial system configuration included an hBN membrane fixed at *z* = 75 Å, with salt water on the feed side and fresh water on the permeate side, all within a simulation box of 160 Å in the *z*-direction (perpendicular to the membrane) and a cross-sectional area of ∼32.649 × 34.8 Å^2^ in the *xy* plane (parallel to the membrane). Rigid graphene pistons were placed at *z* = 5 and 97 Å, as shown in Figure 1a. On the feed side, 2000 water molecules, 22 Na^+^ ions, and 22 Cl^–^ ions were taken, resulting in a salt concentration of 35.7 g/L, which is representative of seawater salinity (∼35 g/L). Additionally, 18 boric acid molecules (∼30.915 g/L) were added to study boron permeation, with a higher concentration than the typical seawater level of ∼4.5 mg/L to improve statistical accuracy. On the permeate side, 500 water molecules were added to model the pure water side of the system.

MD simulations were performed using the LAMMPS package with a 1 fs time step. The nanoporous hBN membrane was allowed to deform in response to forces from water molecules and ions, while atoms farther than 12 Å from the pore center remained fixed. To apply pressure, a constant external average force (*F* = *PA*/*n*) was exerted on each atom in the piston, where *P* is the pressure, *A* is the piston area, and *n* is the number of atoms in the piston. The system was equilibrated in the *NP*_*xy*_ *T* ensemble at 1 atm for 50 ps after energy minimization. During the production run, a pressure of 50 MPa was applied to the feed-side piston and 1 atm to the permeate-side piston (both using the fix aveforce command in LAMMPS) in the *NP*_*xy*_ *T* ensemble. We applied a pressure of 50 MPa to accelerate water transport and obtain statistically meaningful flux within feasible simulation times. Such elevated pressures are commonly used in MD simulations, as water flux scales linearly with applied pressure.^7,14,29,47^ To confirm this, we performed simulations at 25 MPa and 100 MPa pressure for the two best pores used in our study, along with additional simulations at 10 MPa for the P36–N­(3O,9H) pore to examine transport behavior under lower applied pressure conditions closer to practical RO operation (∼5.5–7 MPa^92^), which showed consistent linear scaling (see Section S14). To ensure statistical reliability, we averaged results over six independent simulations with different initial configurations. Error bars indicate the standard error of the mean, computed as σ/√n, with σ representing the standard deviation across the independent runs and n the number of simulations. For the P36–B­(H) and P36–N­(3O,9H) systems, we further conducted ten independent simulations and found the results to be consistent with those from six replicas, confirming that six runs are sufficient for converged flux and salt rejection values, as shown in Section S15. Notably, previous desalination studies often report results based on only one to four replicas.^7,14^ To further assess the convergence and statistical reliability of the reported near-complete rejection behavior, additional convergence analysis for the best-performing nanopores, including cumulative ion permeation events as a function of simulation time across independent replicas together with tabulated crossing statistics, is provided in Section S16.

In the desalination simulations, 200 water molecules (10% of the total on the feed side) were allowed to cross the membrane to the permeate side, minimizing the effect on feed-side salinity. Water permeance, ion rejection, and overall salt rejection were calculated for all the MD simulations conducted for different nanopores. Water permeance was defined as the rate of water flow per unit pressure drop per unit surface area of the membrane. Ion rejection was determined using the formula $\left(1-\frac{N_{{\mathrm{Na}}^{+}/{\mathrm{Cl}}^{-},\mathrm{permeate}}\left(t\right)}{N_{{\mathrm{Na}}^{+}/{\mathrm{Cl}}^{-},\mathrm{feed}}\left(0\right)}\right)\times 100$ , where *N*_Na^+^/Cl^–^,permeate/feed_(*t*) denotes the number of ions present in the permeate/feed side at time *t*. A rejection of 100% indicates no ions passed from the feed to the permeate side. For PMF calculations, water density was averaged using 100,000 samples collected every 20 timesteps over 2 ns for Boltzmann sampling. Note that the driving pressure for water permeance is the net pressure difference (Δ*P* – Δπ), where the osmotic pressure difference in our system is Δπ = 3.026 MPa. In all the considered hBN models, the nanopore concentration (i.e., pore density) in the hBN layer was ∼0.088 nm^–2^. We additionally examined the effect of pore density for the representative high-performance P36–B­(H) pore using a larger membrane model corresponding to a lower pore density of ∼0.0272 nm^–2^. Although the absolute water permeance changes with pore density, the overall transport and rejection trends remain consistent, suggesting that the observed charge-dependent transport characteristics primarily originate from intrinsic pore-level electrostatic interactions (Section S17). Further analysis of the membrane dipole moment, partial charge distribution, and interfacial water polarization revealed that the lower pore-density membrane exhibits stronger electrostatic polarization and more pronounced water ordering near the nanopore (Section S18). This indicates that the conclusions regarding the relationship between edge charge and salt/boron rejection, as well as water permeation, may depend on the nanopore density considered.

## Supplementary Material



## Notes

Water permeance and salt rejection performance with N-terminated P16 pores, discussion on the PMF calculations and their use to rationalize the water flux through various pores, calculated pore diameter for the various nanopores studied here, individual ion rejection data, rescaled partial charge distribution for the various pores used in the study, probing ion interactions with the hBN nanopores’ functional groups and the entire membrane via MD simulations, DFT energy stability analysis of functionalized pore configurations, MD and AIMD simulation results for graphene 2D membrane, radial distribution function (RDF) analysis, lower concentration boric acid simulations, details on the computational modeling and simulations for DFT calculations including the charge rescaling factor employed for the different pores, performance of the P36–B­(6OH,6H) nanopore in seawater desalination with different DDAP parameters, information about the force-field parameters used in the water desalination simulations, impact of applied pressure on the MD simulation outcomes for P36–N­(3O,9H) and P36–B­(H) nanopores, impact of number of replicas considered while averaging the MD simulation outcomes for P36–N­(3O,9H) and P36–B­(H) nanopores, convergence analysis of ion rejection across independent simulations, effect of pore density on water transport and salt rejection, and effect of pore density on water structuring, dipole orientation, and dipole ordering (PDF)
