# Supplementary material for: Desalination Design Rules for Nanoporous 2D Materials: Low Edge Charges Sieve Ions, While Positive Edge Charges Exclude Boron
Source: ACS Mater Au. 2026 Aug 31;6(6):1337–51. doi: 10.1021/acsmaterialsau.6c00074 (PMC13760655; doi:10.1021/acsmaterialsau.6c00074)
Supplement: Supplementary file 1 [file mg6c00074_si_001.pdf]

Supporting Information for:

Desalination Design Rules for Nanoporous 2D Materials: Low Edge Charges Sieve Ions, While Positive Edge Charges Exclude Boron

Pradeep Dhondi, Sagar Ghorai, and Ananth Govind Rajan*

Department of Chemical Engineering, Indian Institute of Science, Bengaluru, Karnataka 560012, India

*Email: ananthgr@iisc.ac.in

Table of Contents

S1. Performance of P16-N and P16-N(H) nanopores in seawater desalination 2

S2. Rationalizing the relative water flux through various pores through free energy calculations... 2

S3. Determination of the size of various pore models used in this study 3

S4. Individual ion rejection data 5

S5. Partial charges on B and N atoms in various nanoporous hBN configurations.............. 7

S6. Probing ion interactions with nanoporous hBN via potential energy calculations 9

S7. DFT energy stability analysis of functionalized pore configurations 12

S8. MD and AIMD simulation results for functionalized graphene 2D membranes............ 13

S9. Radial distribution function (RDF) analysis 16

S10. Lower concentration boric acid simulations 17

S11. Details of the density functional theory calculations used to determine the partial charges on the 2D sheets 17

S12. Performance of P36-B(6OH,6H) nanopore in seawater desalination with different DDAP parameters 20

S13. Table of the force-field parameters taken from previous work................................. 21

S14. Impact of applied pressure in the MD simulation outcomes for P36-N(3O,9H) and P36-B(H) nanopores 22

S15. Impact of the number of replicas while averaging the MD simulation outcomes for P36-B(H) and P36-N(3O,9H) nanopores 22

S16. Convergence analysis of ion rejection across independent simulations 23

S17. Effect of pore density on water transport and salt rejection 24

S18. Effect of pore density on water structuring, dipole orientation, and dipole ordering . 25

S1. Performance of P16-N and P16-N(H) nanopores in seawater desalination

Table S1. Water permeance and salt rejection performance with an N-terminated P16 pore.

	P16-N	P16-N(H)
**Water permeance ($L\ cm^{-2}\ MPa^{-1}\ day^{-1}$)**	25.6 ± 2.0	2.7 ± 0.2
**Salt rejection (%)**	99.1 ± 0.5	100 ± 0.0

S2. Rationalizing the relative water flux through various pores through free energy calculations

To understand the water permeance trend and ion rejection through different nanopores, we calculated the one-dimensional (1D) potential of mean force (PMF) as $F(z) = -RT\ln[\rho(z)]$, where R is the universal gas constant (R = 8.314 J mol^{-1} K^{-1}), T is the temperature of the system (298.15K), $\rho(z)$ is the water density at position z, calculated by including only water molecules within a cylindrical region of radius 9 Å centered on the nanopore axis. This spatial restriction ensures that the analysis captures only water molecules translocating through the pore while minimizing contributions from the excluded volume of the 2D material sheet. $F(z)$ represents the free energy profile for water molecules passing through the nanopores. As shown in Figure S1, the PMF plots are consistent with the water permeance for the various nanopores investigated here and follow the order P36-N < P25-N < P16-N ≈ P36-N(H) < P25-N(H) < P16-N(H) for the N-terminated pores and P36-B < P25-B < P36-B(H) < P25-B(H) for the B-terminated pores. Indeed, our simulations reveal that the free energy barrier for the P16-N(H) is the highest resulting in low water permeance through it. This trend aligns with Gao et al.'s MD simulations[1] with unfunctionalized 2D hBN membranes, which showed that N-terminated pores had higher water permeance but lower salt rejection than B-terminated pores of similar size.

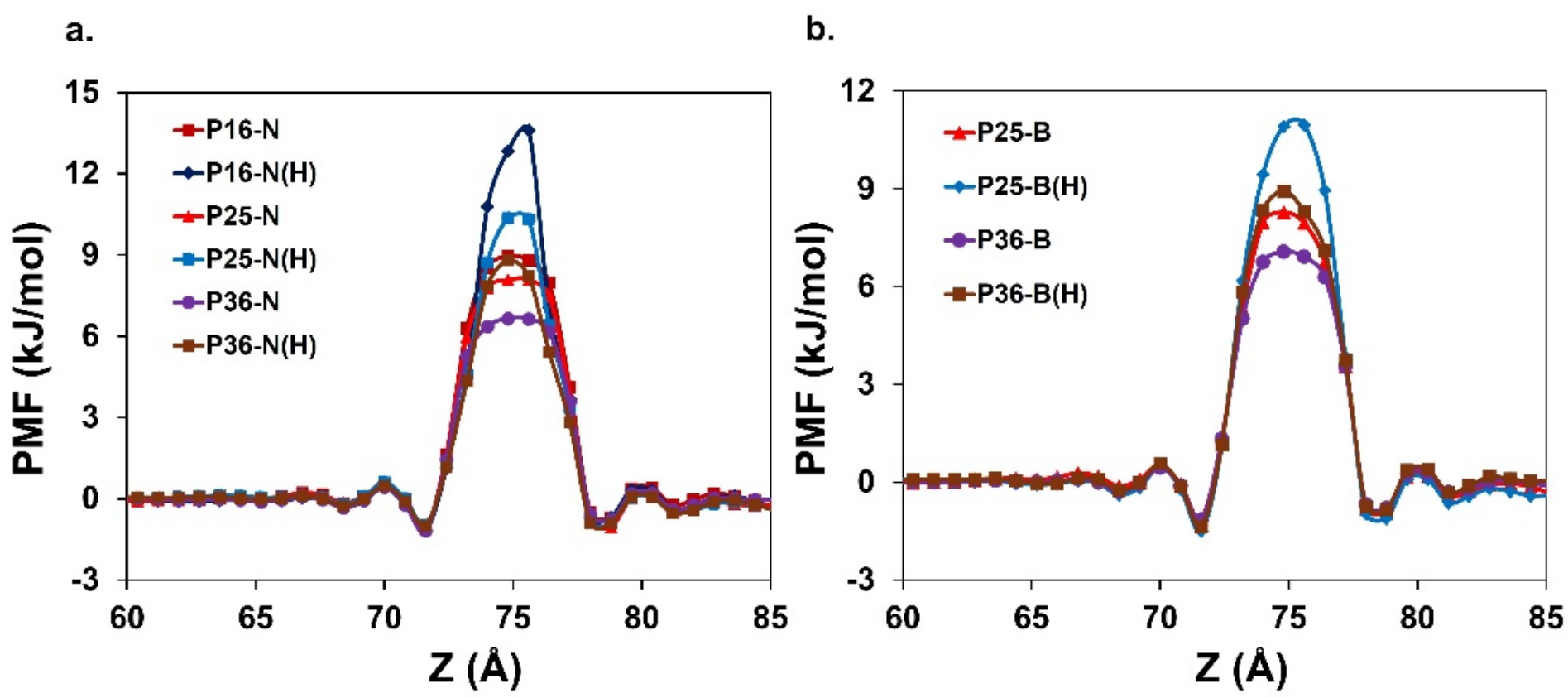

Figure S1. The potential of mean force (PMF) profiles along the direction perpendicular to the membrane (labelled the Z axis) for water flow through various nanoporous hBN membranes. (a,b) PMF for NaCl solution permeating through H-functionalized (a) N- and (b) B-terminated pores. The trends in PMF correlate with water permeance, following the order P36-N < P25-N < P16-N ≈ P36-N(H) < P25-N(H) < P16-N(H) for N-edge pores and P36-B < P25-B < P36-B(H) < P25-B(H) for B-edge pores.

S3. Determination of the size of various pore models used in this study

a.

N and B terminated pores	Average pore limiting diameter (Å)
P16-N	4.87
P25-N	5.98
P36-N	7.64
P16-N(H)	4.15
P25-N(H)	4.93
P36-N(H)	6.41
P36-N(3O,9H)	4.85
P25-B	5.63
P36-B	7.12
P25-B(H)	4.00
P36-B(H)	5.8
P36-B(2OH,10H)	5.78
P36-B(6OH,6H)	5.03

b.

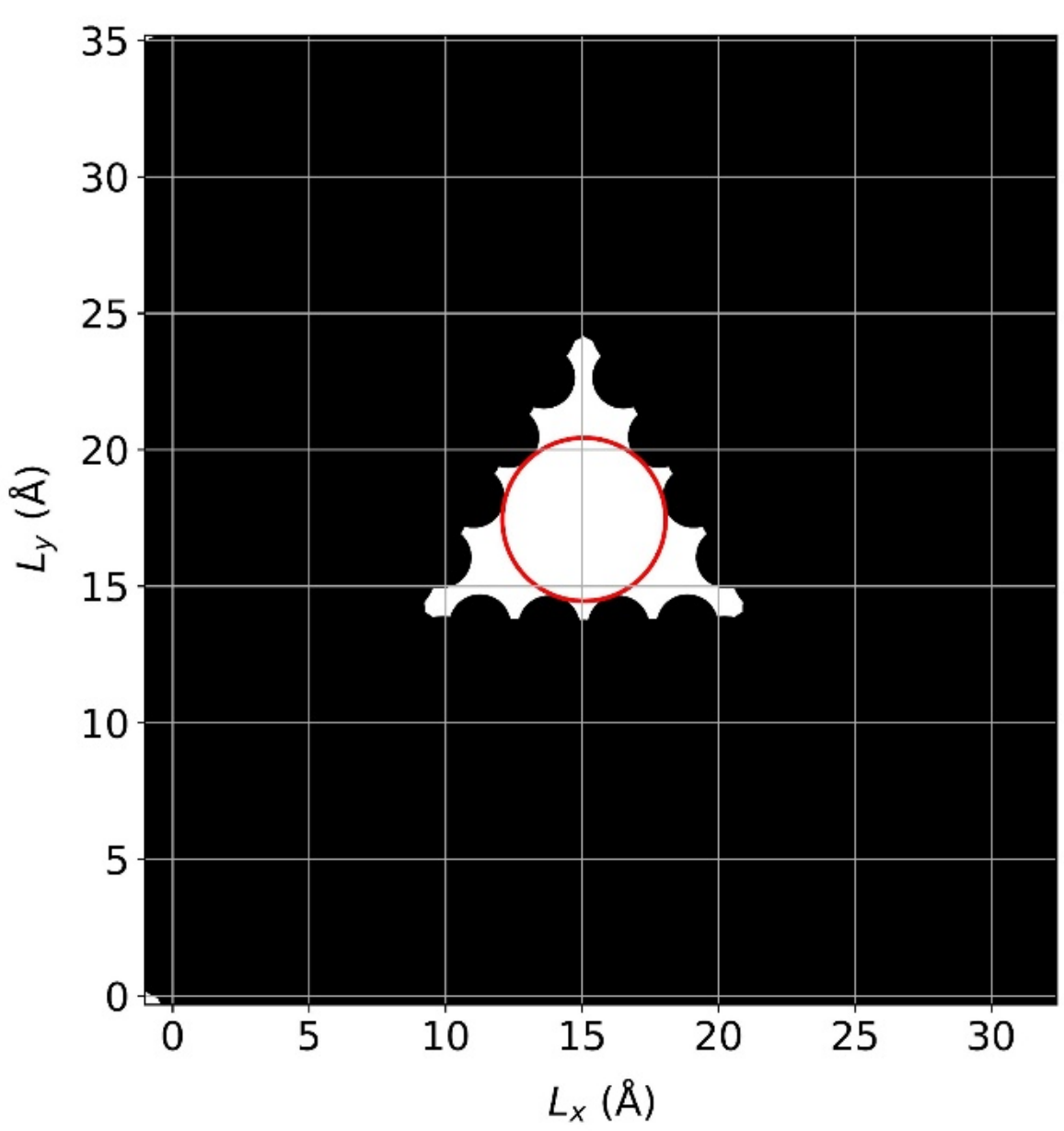

Figure S2. Calculation of the largest van der Waals radius that fits in the various nanopores considered. (a) Table of the average pore limiting diameter for different nanoporous hBN models with unfunctionalized and functionalized N and B-terminated pores. (b) Visualization of the pore with the maximum van der Waals sphere inscribed using a custom Python code. The red circle indicates the maximum van der Waals sphere inscribed. L_x (Å) and L_y (Å) denote the position along the hBN sheet in the x and y directions, respectively. The van der Waals radii for each atom type (B, N, O, H) are taken from Mantina et al.[2]

To estimate the nanopore area for each pore, the B, N, O, and H atoms were taken into consideration as discs with a radius equal to the van der Waals radius of the corresponding atom (i.e., 1.92 Å for B, 1.55 Å for N, 1.10 Å for H, and 1.52 Å for O).[2] After excluding B, N, O, and H atom discs, the radius of the largest circle that could be inscribed in the nanopore was determined, as shown in Figure S2.

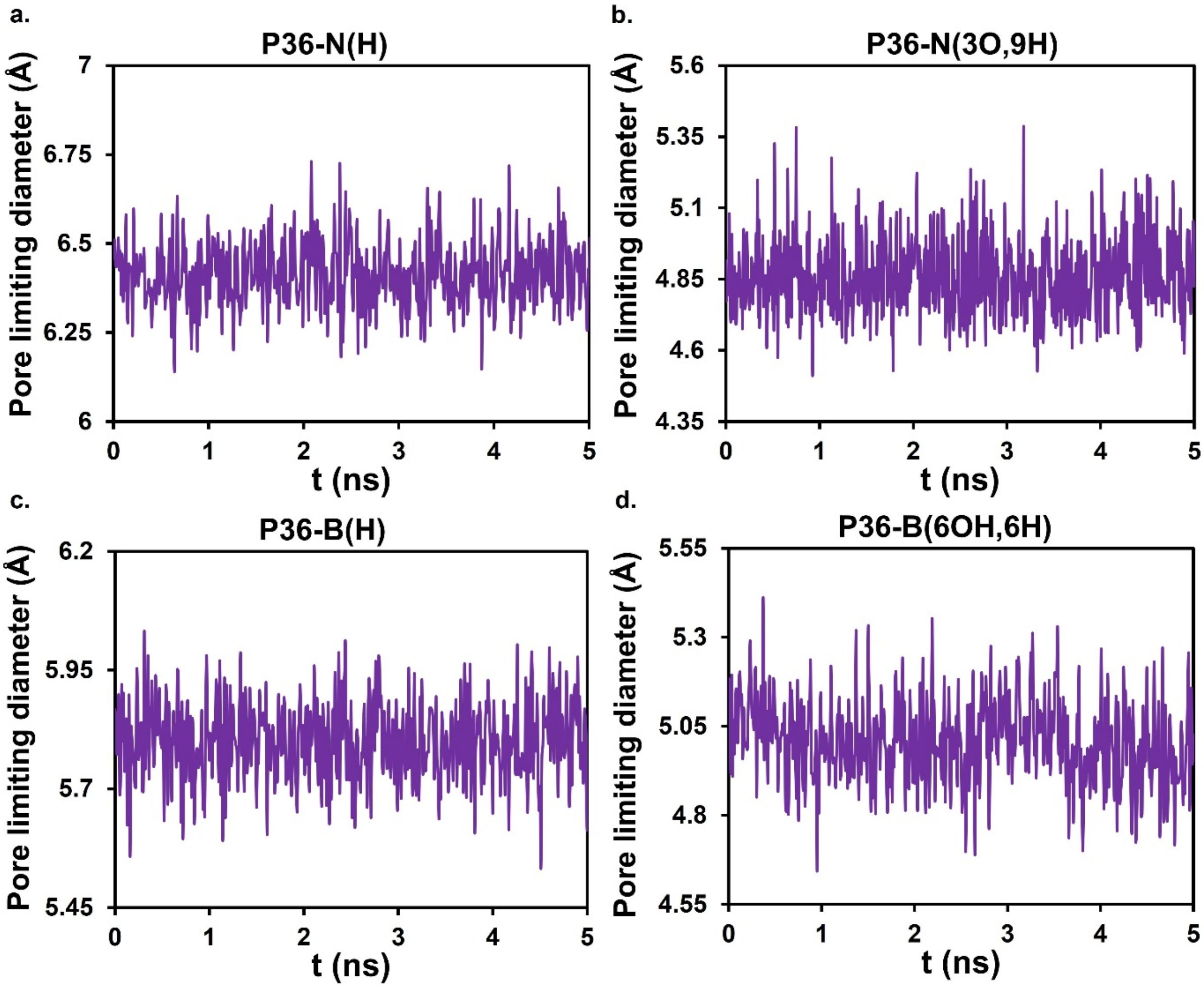

Figure S3. Pore limiting diameter as a function of simulation time for the (a) P36-N(H), (b) P36-N(3O,9H), (c) P36-B(H), and (d) P36-B(6OH,6H) nanopores.

S4. Individual ion rejection data

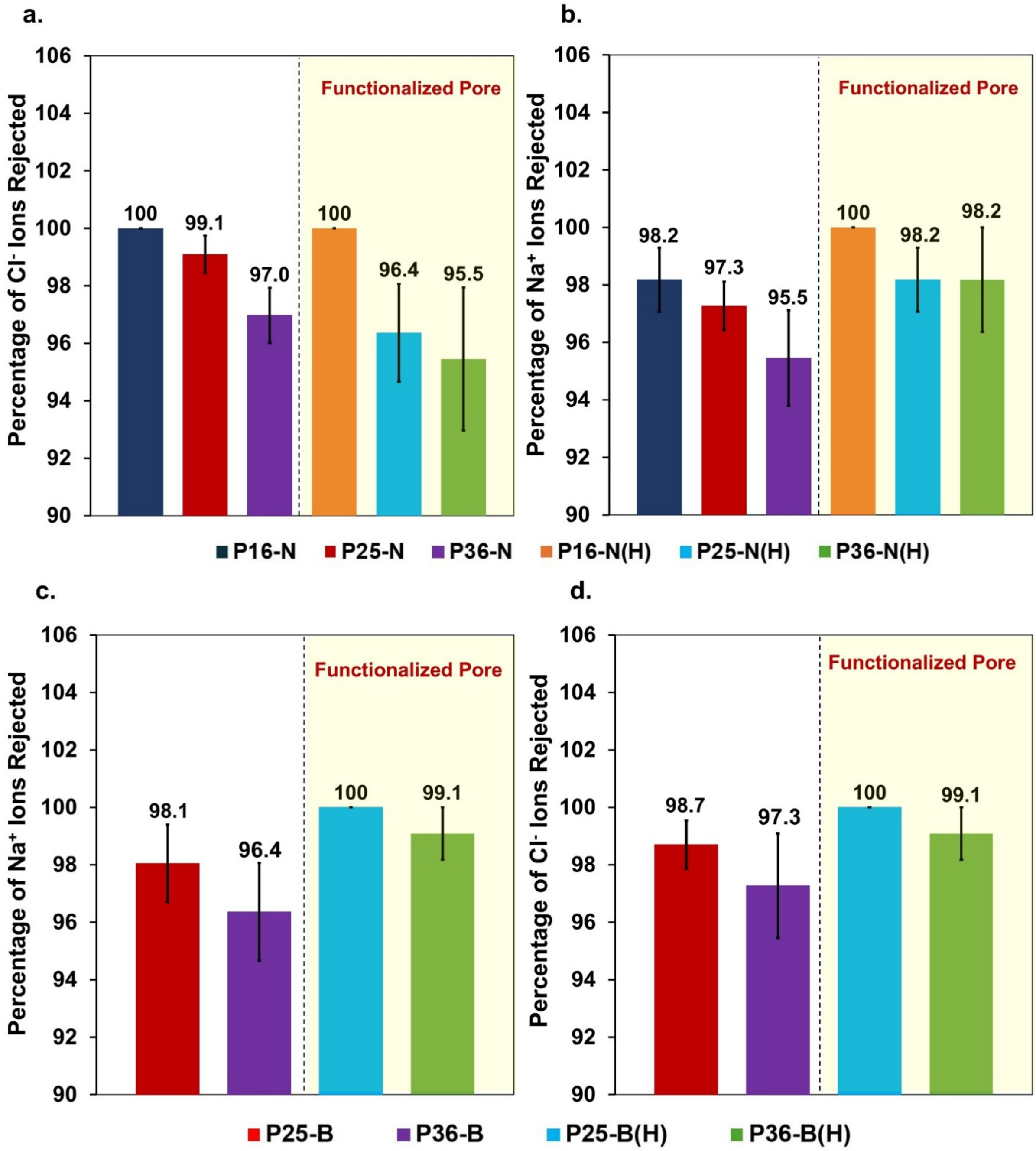

Figure S4. MD simulation results for the separation of NaCl aqueous salt solution through nanopores of varying sizes and edge terminations with H functionalization. (a,b) Percentage of Na^+/Cl^- ions passing from the feed side to the permeate side through N-terminated pores. (c,d) Percentage of Na^+/Cl^- ions passing from the feed side to the permeate side through B-terminated pores. In all hBN models, the nanopore density was ~0.088 nm^{-2}. The results are divided into two regions, with a black dashed line distinguishing functionalized and unfunctionalized pores. Hydrogen-functionalized nitrogen (N) and boron (B) terminated pores are highlighted with a light yellow background, while unfunctionalized pores are presented with a white background. Note that the data for the P36 pores are adapted from Ghorai et al.[3]

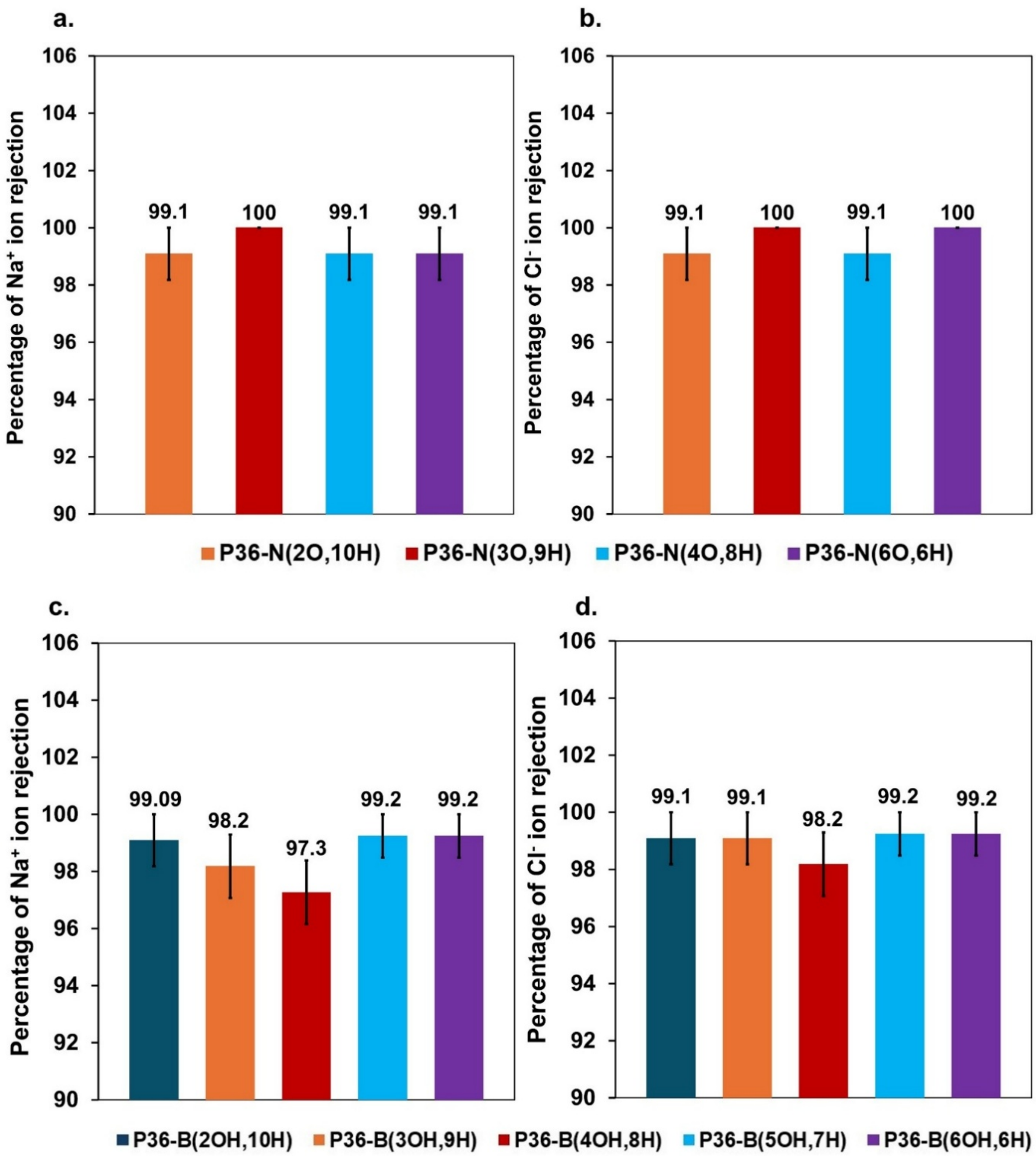

Figure S5. MD simulation results for the separation of NaCl aqueous salt solution through nanopores of varying sizes and edge terminations with OH/O/H functionalization. (a,b) Percentage of Na^+/Cl^- ions passing from the feed side to the permeate side through N-terminated pores. (c,d) Percentage of Na^+/Cl^- ions passing from the feed side to the permeate side (100% signifies no ions permeated in four simulations conducted with different initial configurations of ions and water in the system) through a B-terminated pore. In all hBN models, the nanopore density was ~0.088 nm^{-2}.

S5. Partial charges on B and N atoms in various nanoporous hBN configurations

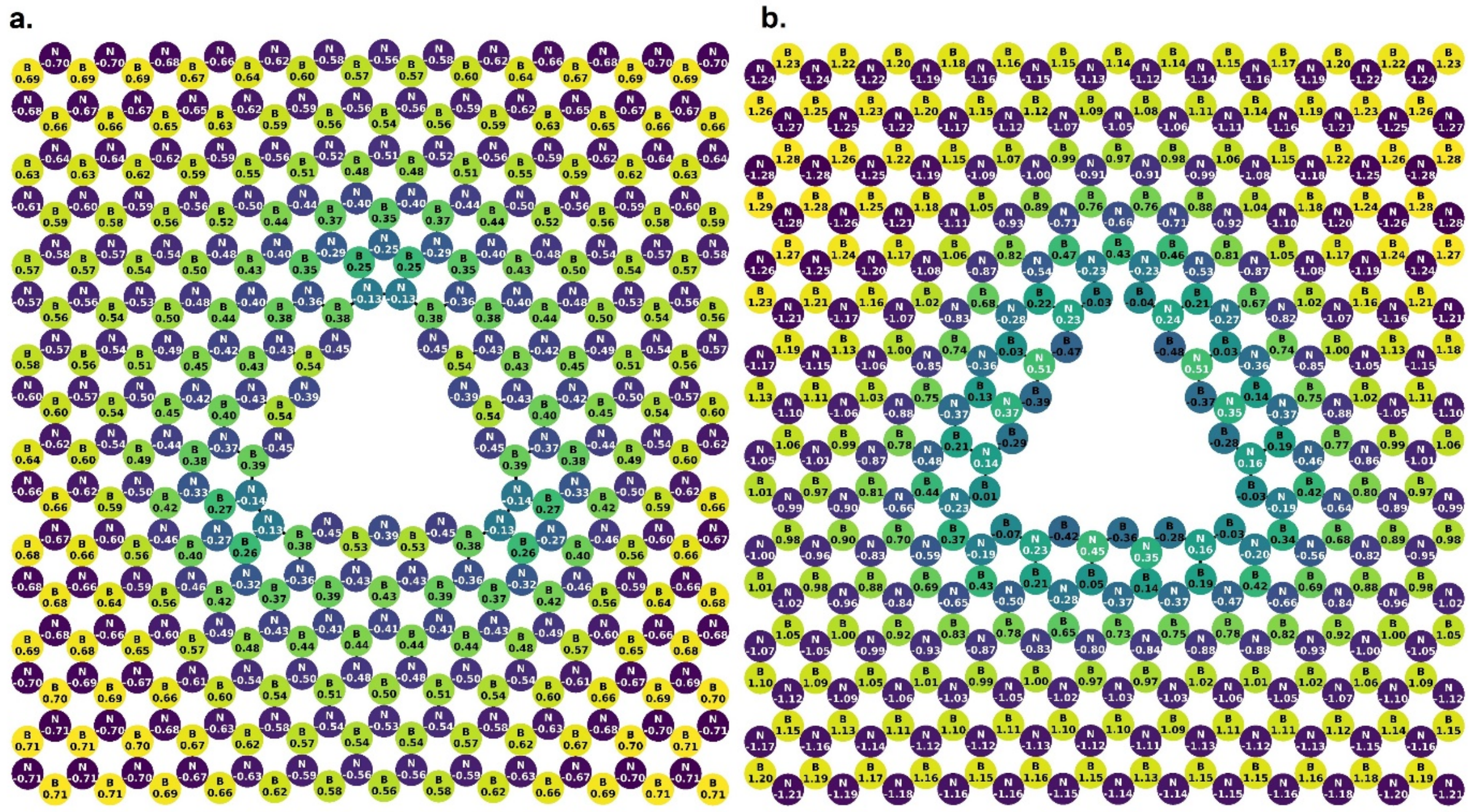

Figure S6. Rescaled partial charge distribution (in elementary charge units, e) computed using the default DDAP parameters for (a) the P25-N pore and (b) the P25-B pore. In all hBN models, the nanopore density was ~0.088 nm^{-2}.

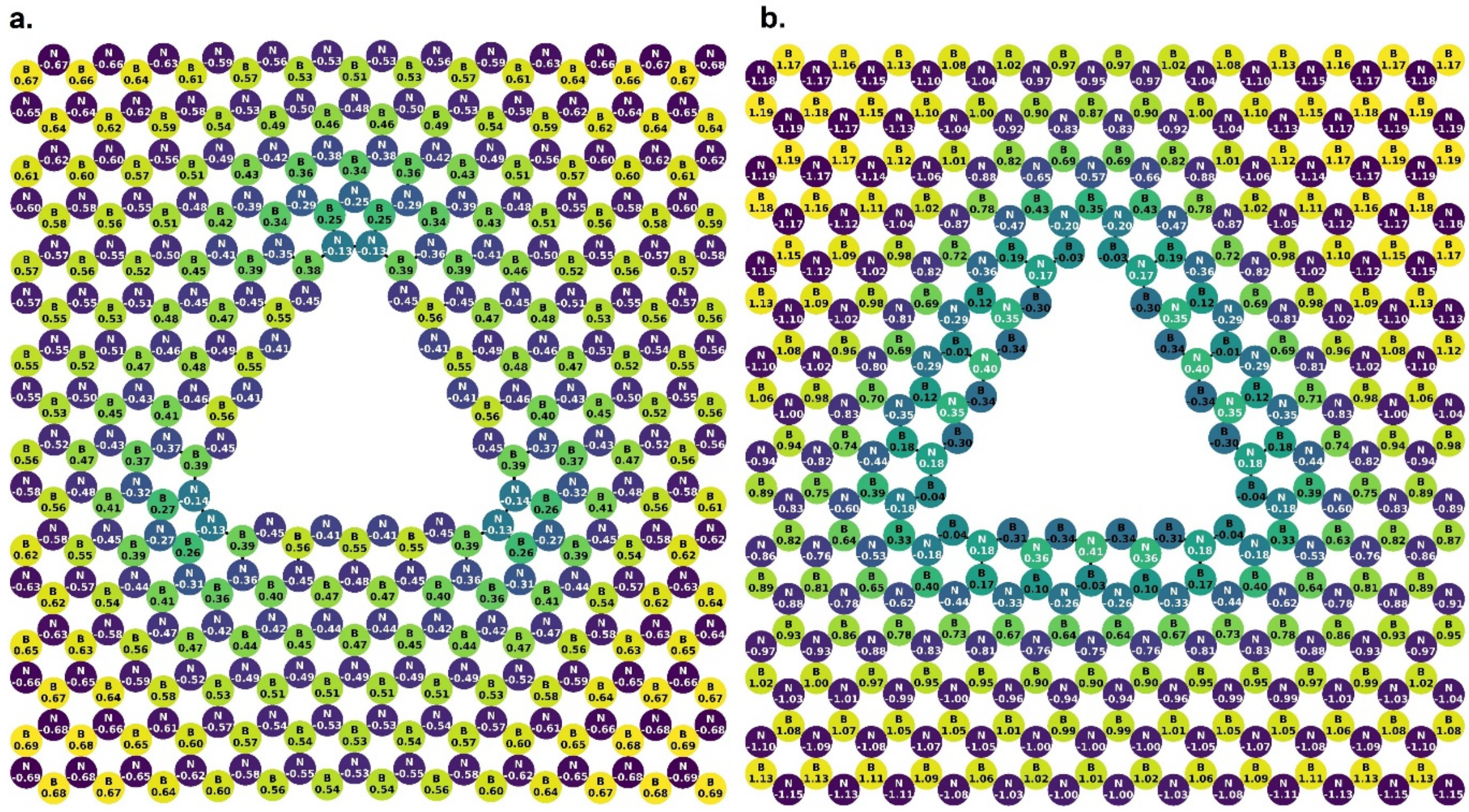

Figure S7. Rescaled partial charge distribution (in elementary charge units, e) computed using the default DDAP parameters for (a) the P36-N pore and (b) the P36-B pore. In all hBN models, the nanopore density was ~0.088 nm^{-2}. This image showing the charge distribution for the P36 pore is adapted from Ghorai et al.[3] Copyright 2025, American Institute of Physics.

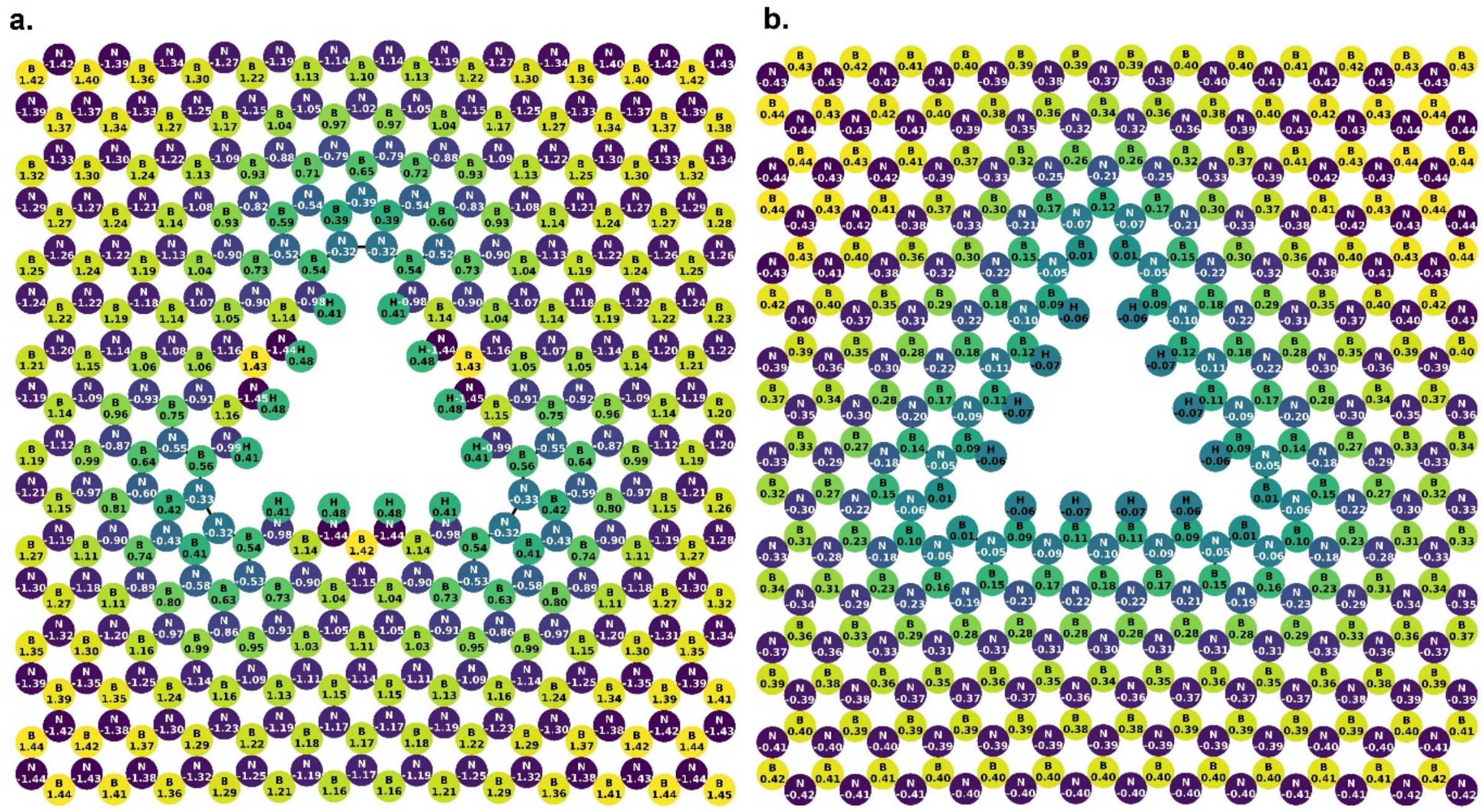

Figure S8. Rescaled partial charge distribution (in elementary charge units, e) computed using the default DDAP parameters for (a) the P36-N(H) pore and (b) the P36-B(H) pore. In all hBN models, the nanopore density was ~0.088 nm^{-2}. This image showing the charge distribution for the P36 pore is adapted from Ghorai et al.[3] Copyright 2025, American Institute of Physics.

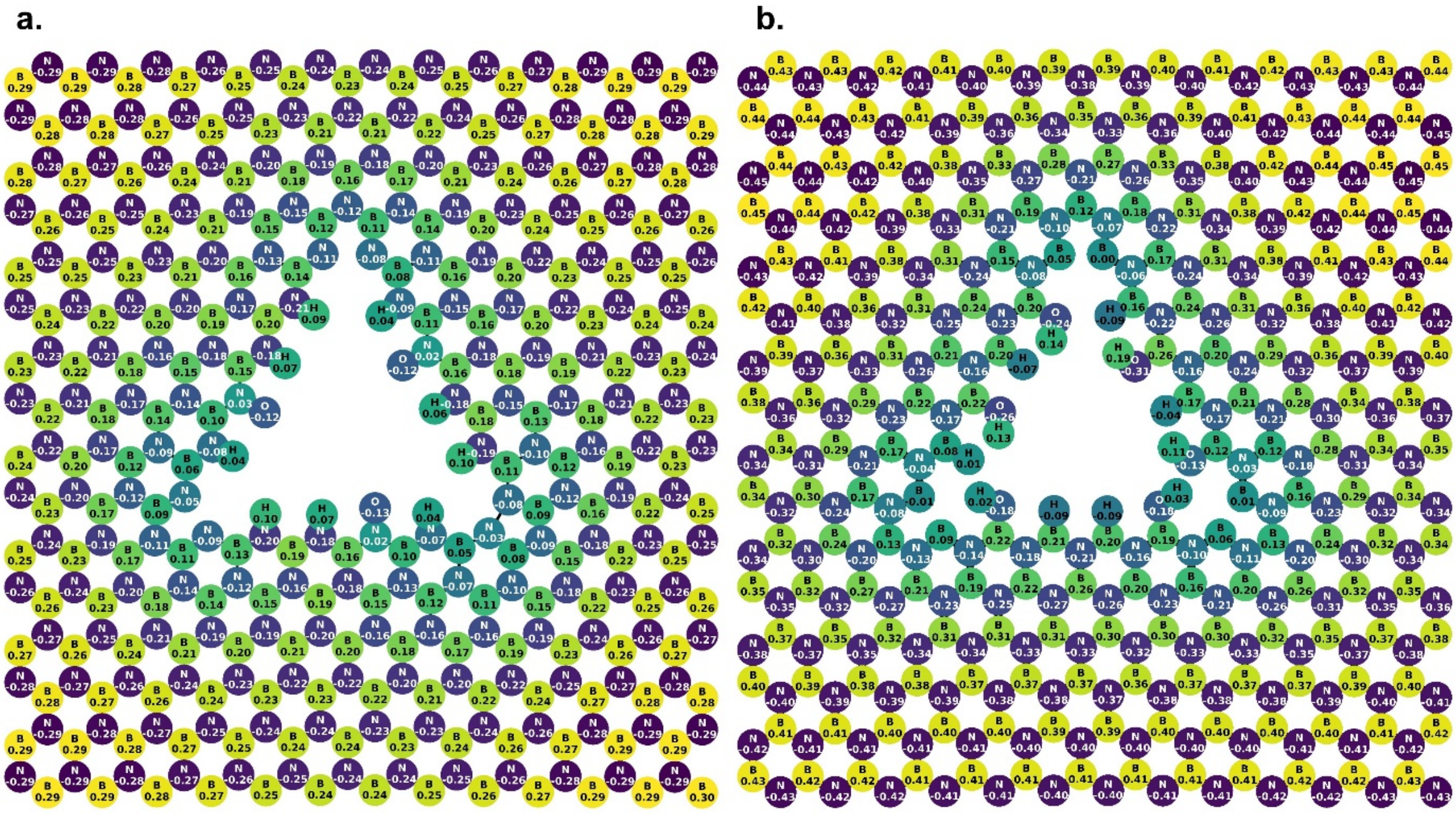

Figure S9. Rescaled partial charge distribution (in elementary charge units, e) computed using the default DDAP parameters for (a) the P36-N(3O,9H) pore and (b) the P36-B(6OH,6H) pore. In all hBN models, the nanopore density was ~0.088 nm^{-2}.

S6. Probing ion interactions with nanoporous hBN via potential energy calculations

We conducted molecular dynamics simulations to evaluate the Lennard-Jones (LJ) and Coulombic (Coul) energy contributions between ions and the nanoporous hBN membrane. The system containing 2000 water molecules, 22 Na^+ ions, and 22 Cl^- ions, was placed on either side of the nanoporous hBN membrane. The simulation box measured ~32 × 34 Å^2 in the *xy*-plane and extended 260 Å along the *z*-axis, with the hBN sheet fixed at z = 75 Å. Graphene pistons were used at the *z*-boundaries to maintain ~1 atm pressure and establish equilibrium conditions. To compute energy profiles, a single Na^+ or Cl^- was selected, fixed at discrete positions along the z-axis (65-85 Å, spacing 1 Å), and equilibrated for 2 ns in each configuration. The time-averaged total potential energy (TPE), along with its LJ and Coulombic components, was calculated by rerunning the trajectories in LAMMPS using the compute group/group command with the "pair yes" and "kspace yes" options, where TPE is the sum of LJ and Coulombic interactions. Note that both groups used in the group/group command for ion-pore edge interactions carry net charges, and the calculated interactions reflect these charge contributions.

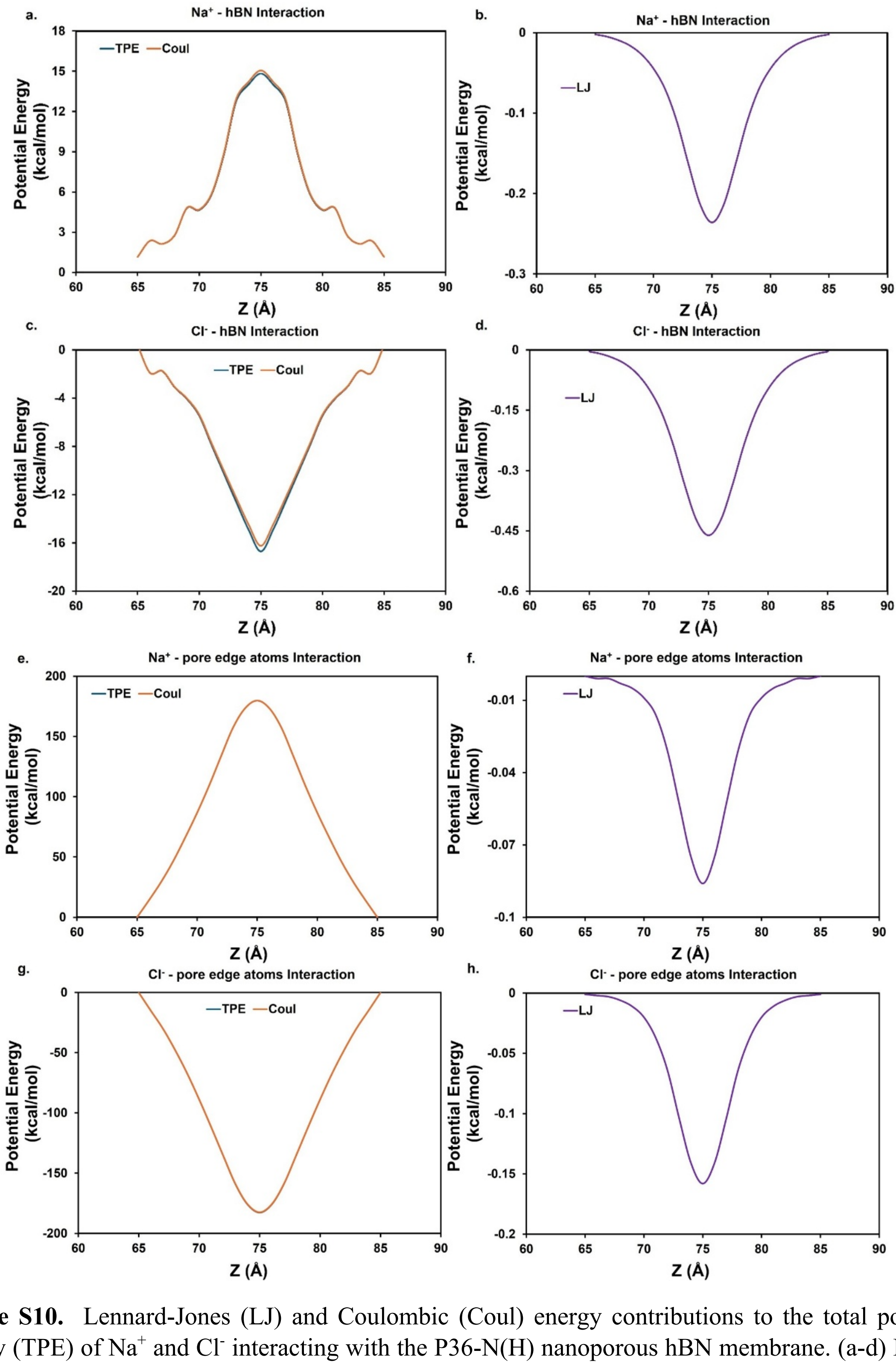

Figure S10. Lennard-Jones (LJ) and Coulombic (Coul) energy contributions to the total potential energy (TPE) of Na^+ and Cl^- interacting with the P36-N(H) nanoporous hBN membrane. (a-d) Energy landscapes for Na^+ (a-b) and Cl^- (c-d) interacting with the entire hBN sheet, decomposed into TPE, LJ, and Coul components as a function of distance (*z*). (e-h) Corresponding energy profiles for Na^+ (e-f) and Cl^- (g-h) interacting specifically with the functionalized pore-edge atoms. TPE is computed as the sum of LJ and Coulombic interactions. Note that ion-pore edge interactions are modified such that the interaction energy is reduced from its absolute value to approach zero at z = 65 Å.

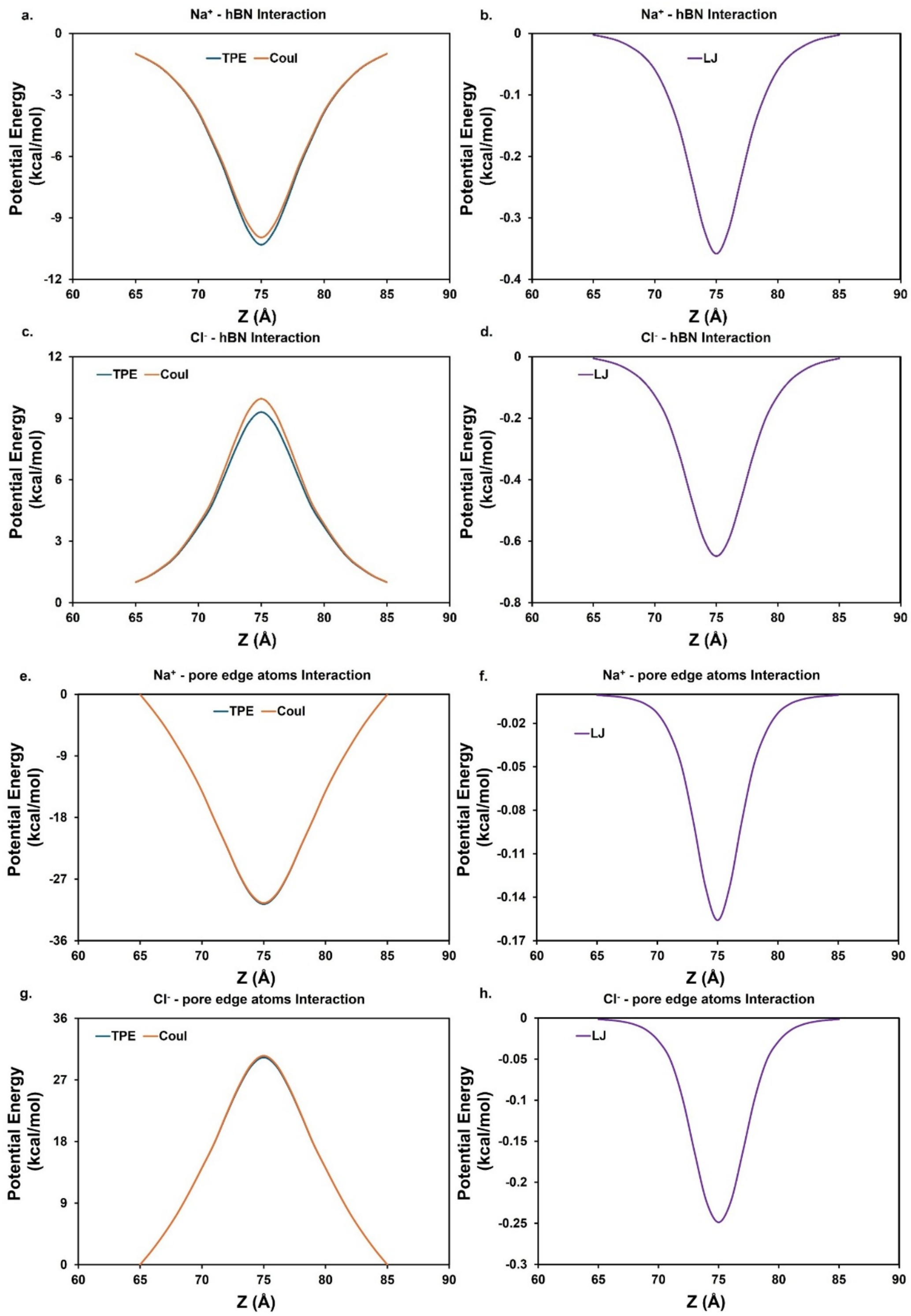

Figure S11. Lennard-Jones (LJ) and Coulombic (Coul) energy contributions to the total potential energy (TPE) of Na^+ and Cl^- interacting with the P36-B(H) nanoporous hBN membrane. (a-d) Energy landscapes for Na^+ (a-b) and Cl^- (c-d) interacting with the entire hBN sheet, decomposed into TPE, LJ, and Coul components as a function of distance (*z*). (e-h) Corresponding energy profiles for Na^+(e-f) and Cl^- (g-h) interacting specifically with the functionalized pore-edge atoms. TPE is computed as the sum of LJ and Coulombic interactions. Note that ion-pore edge interactions are modified such that the interaction energy is reduced from its absolute value to approach zero at z = 65 Å.

S7. DFT energy stability analysis of functionalized pore configurations

a.

Type	Reaction	Relative formation energy per functionalized atom (eV)
P36-B(6OH,6H) – A	$X + 6H_2O \rightarrow X(OH)_6H_6$	-2.12
P36-B(6OH,6H) – B	$X + 6H_2O \rightarrow X(OH)_6H_6$	-2.1

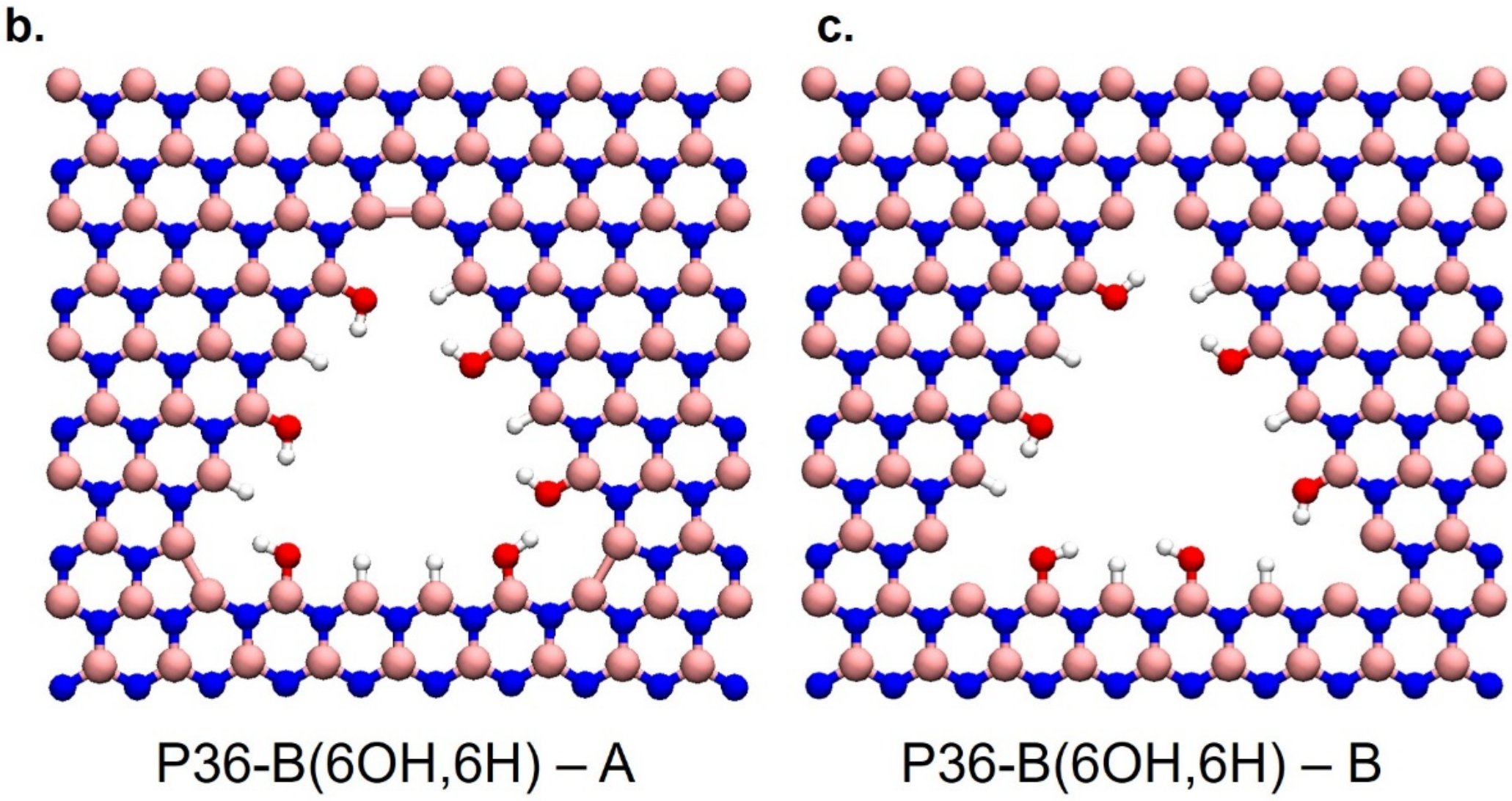

Figure S12. DFT energy comparison of different functionalized configurations for the P36-B(6OH,6H) hBN nanopore. (a) Tabulated relative formation energies per functionalized atom for the two representative pore configurations. (b,c) Optimized atomic structures corresponding to the P36-B(6OH,6H)-A and P36-B(6OH,6H)-B configurations, respectively. The slightly-lower-energy P36-B(6OH,6H)-A configuration was employed in the MD simulations and is consistent with the stable edge-functionalized structure observed in AIMD simulations.[3] The DFT reference energies for a water molecule were obtained by modeling an isolated H_2O unit within a sufficiently large vacuum box to minimize spurious periodic interactions. All DFT calculations described above were performed using the CP2K package.

S8. MD and AIMD simulation results for functionalized graphene 2D membranes

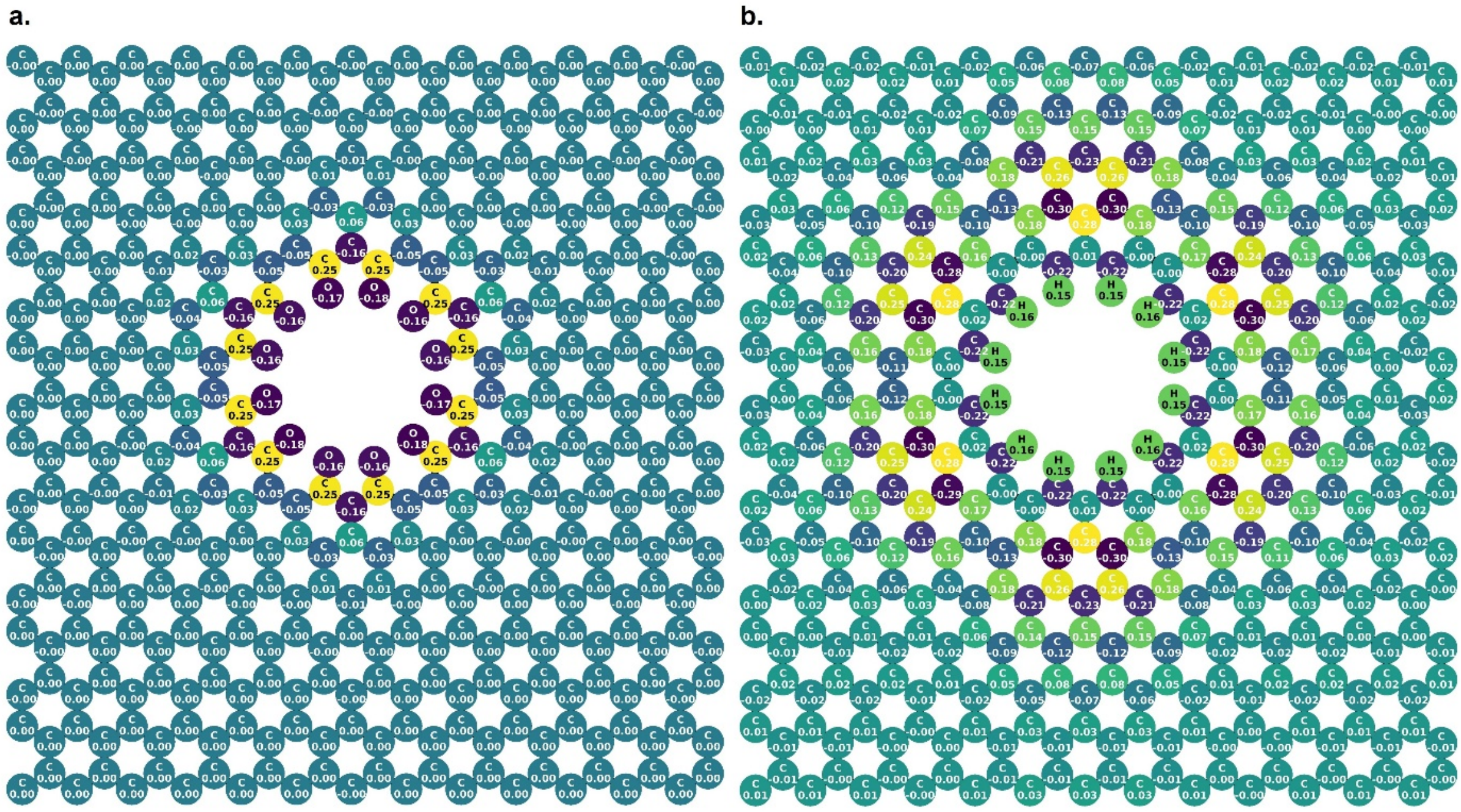

Figure S13. Rescaled partial charge distribution (in elementary charge units, e) computed using the DDAP parameters for (a) the P24-C(O) pore and (b) the P24-C(H) pore. In all graphene models, the nanopore density was ~0.09 nm^{-2}.

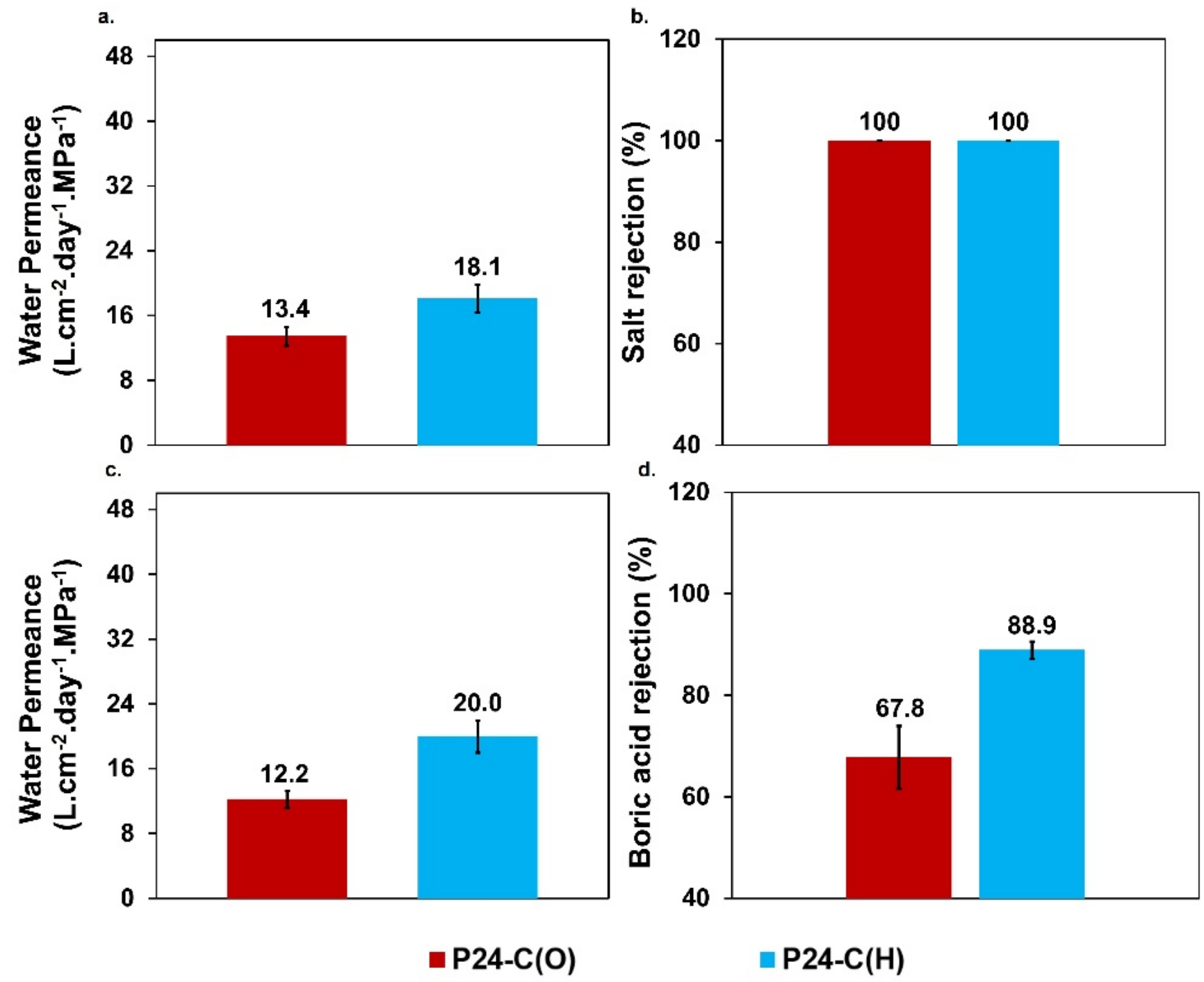

Figure S14. MD simulation results for the separation of NaCl aqueous solution and boron rejection through graphene nanopores with O/H edge functionalization. P24-C(H) denotes a pore created by removing 24 carbon atoms and functionalizing its edges with 12 hydrogen atoms; P24-C(O) denotes a pore functionalized with 12 oxygen atoms. (a,c) Water permeance (L cm^{-2} day^{-1} MPa^{-1}) from the feed

to the permeate side for (a) NaCl solution and (c) boric acid. (b) NaCl rejection percentage. (d) Boric acid rejection percentage across functionalized graphene nanopores.

Table S2. Diameter of the largest van der Waals sphere that fits different nanoporous graphene models with functionalized O and H-terminated pores.

C terminated pores	Average pore limiting diameter (Å)
P24-C(12O)	4.91
P24-C(12H)	5.96

Note that in the MD simulations, the pore size was kept identical to that of the hBN membrane to enable direct comparison of performance between the two materials.

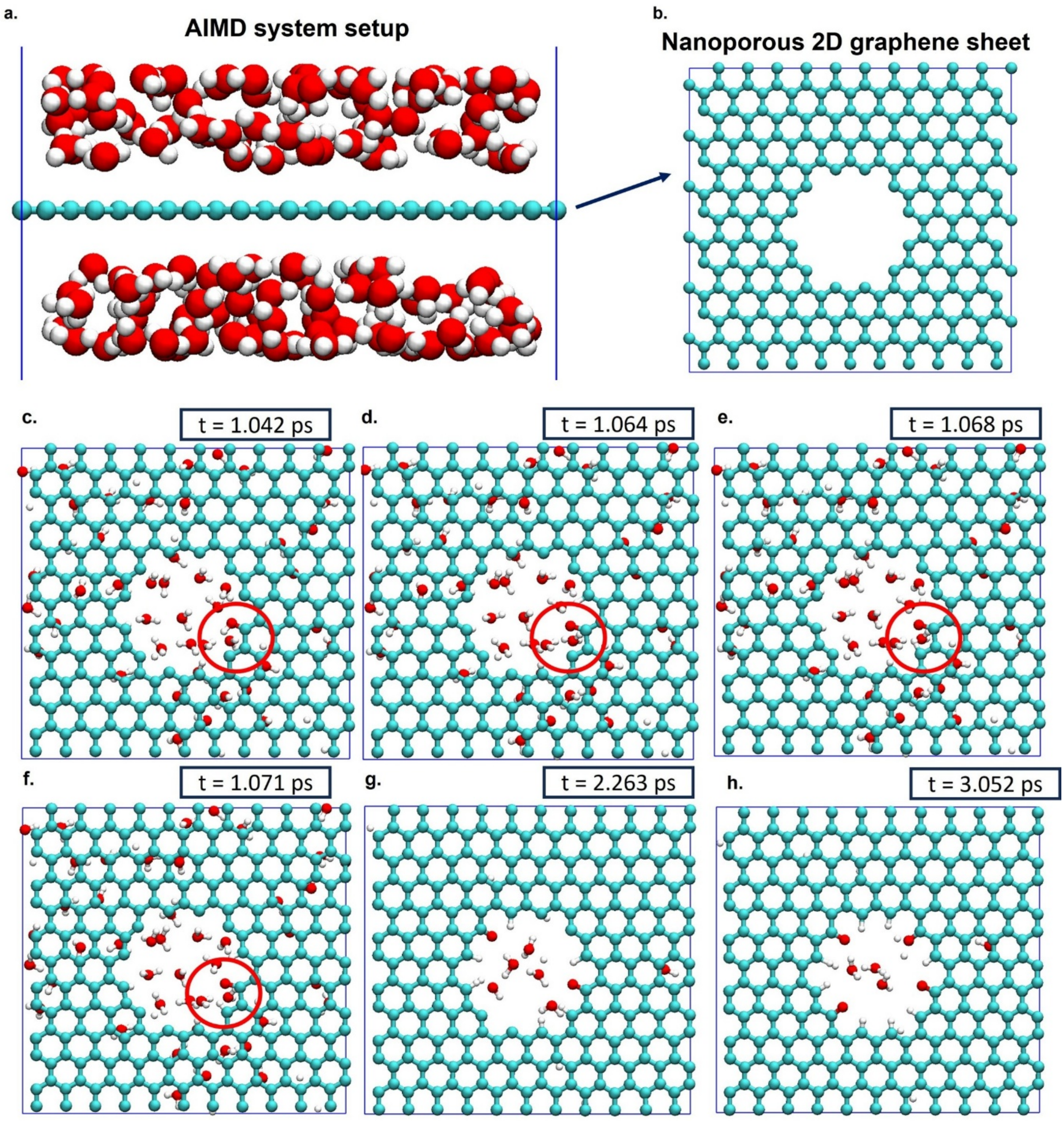

Figure S15. AIMD simulation setup and representative snapshots illustrating the evolution of edge functionalization in a 2D graphene nanopore. (a) AIMD simulation setup. (b) Nanoporous graphene configuration used in the AIMD simulations. (c-h) Time-resolved AIMD snapshots showing the evolution of graphene pore-edge functionalization under aqueous conditions. The simulation time corresponding to each snapshot is indicated in the top-right corner. For clarity, only C, O, and H atoms within the region 13 Å < *z* < 17 Å are shown to highlight the edge functional groups, with the graphene membrane placed around *z* = 15.5 Å. Key structural transitions are highlighted using red circles. Color code: C, cyan; H, white; O, red.

The ab initio molecular dynamics (AIMD) simulations were performed using a simulation box with a length of 45 Å along the perpendicular (*z*) direction and periodic boundary conditions in the lateral *xy* plane, with dimensions of 27.148 × 25.65 $Å^2$. AIMD simulations were conducted using the Vienna Ab initio Simulation Package (VASP), version 6.2.0.[4–6] All electronic structure calculations were carried out using the projector augmented wave (PAW) method[7,8] within a plane-wave basis set, employing a kinetic energy cutoff of 500 eV. The self-consistent field procedure was converged to an energy tolerance of 10^{-5} eV. Exchange-correlation interactions were treated using the generalized gradient approximation in the Perdew-Burke-Ernzerhof (PBE) form.[9] Electronic occupancies were treated using Gaussian smearing with a width of 0.01 eV. Long-range van der Waals interactions were incorporated through Grimme's D3 correction.[10] AIMD were performed in the canonical (NVT) ensemble at 298.15 K, with the number of particles and simulation volume held fixed throughout. Temperature was regulated using a Nosé-Hoover thermostat.[11,12] Atomic motion was propagated using the leap-frog integration scheme with a time step of 1.0 fs. All production simulations were conducted without spin polarization. The graphene sheet was fixed at *z* = 15.5 Å, and 75 water molecules were placed on each side of the membrane with an initial separation of 2 Å from the graphene surface (Figure S15a). A hexagonal graphene nanopore formed by removing 24 carbon atoms was considered in the present study (Figure S15b). The AIMD simulations reveal the spontaneous functionalization of the graphene nanopore edges under aqueous conditions. At approximately 1.042 ps, a water molecule from the bulk adsorbed onto an edge carbon atom without dissociation (Figure S15c). Subsequently, at around 1.064 ps, the adsorbed water molecule underwent proton transfer to a neighboring water molecule, forming H_3O^+, while the remaining oxygen atom bound to the edge carbon atom to form a C=O functional group (Figure S15d). Further snapshots at 1.068 ps and 1.071 ps show proton-transfer-mediated edge functionalization at neighboring sites, where H_3O^+ transfers a proton to nearby edge carbon atoms, resulting in C-H bond formation. This proton transport process is consistent with the Grotthuss mechanism previously discussed in our studies on hBN and MoS_2 nanopores.[3,13] Over the course of the ~3 ps AIMD simulation, the graphene nanopore evolves into a mixed edge-functionalized structure containing four C=O groups and eight C-H terminations (Figure S15h).

S9. Radial distribution function (RDF) analysis

Understanding the hydration structure of molecules and ions is crucial in nanoscale separation and nanopore transport, as it directly impacts water flux and molecular/ionic selectivity. In aqueous environments, ions are surrounded by structured layers of water molecules, thereby forming a hydration shell through electrostatic attraction between the ion and the polar water molecules. The properties of these shells, such as their size, stability, and degree of ordering, govern how easily the ions and water move through confined spaces, especially when external pressure is applied to the system. A key method for characterizing these interactions is the radial distribution function (g(*r*)), which measures the probability of finding a water molecule at a distance *r* from a given ion/molecule. Sharp, well-defined peaks in g(*r*) indicate strong, localized ion/molecule-water interactions and tightly bound hydration shells, while broader or flatter peaks suggest weaker or more diffuse hydration. In our study, g(*r*) was calculated using the compute rdf command in LAMMPS to quantify ion/boric acid-water structuring (Figure S16). Mathematically, g(*r*) is defined as: $\frac{1}{4\pi\rho r^2}\frac{\mathrm{d}N(r)}{\mathrm{d}r}$ where ρ is the average number density of particles and d*N*(*r*) is the number of particles found in a spherical shell of thickness d*r* from the reference particle. Classical molecular dynamics simulations were conducted in a cubic simulation box of dimensions 40 × 40 × 40 Å^3, containing 2000 water molecules along with 22 NaCl units and 18 boric acid molecules. Simulations were performed using LAMMPS under NPT ensemble conditions, with a 1 ns equilibration followed by a 4 ns production run. A timestep of 1 fs was employed during the simulation run. The equilibrated configurations were used to calculate radial distribution functions (RDFs) between selected ion-water and solute-water atom pairs to analyze hydration structure.

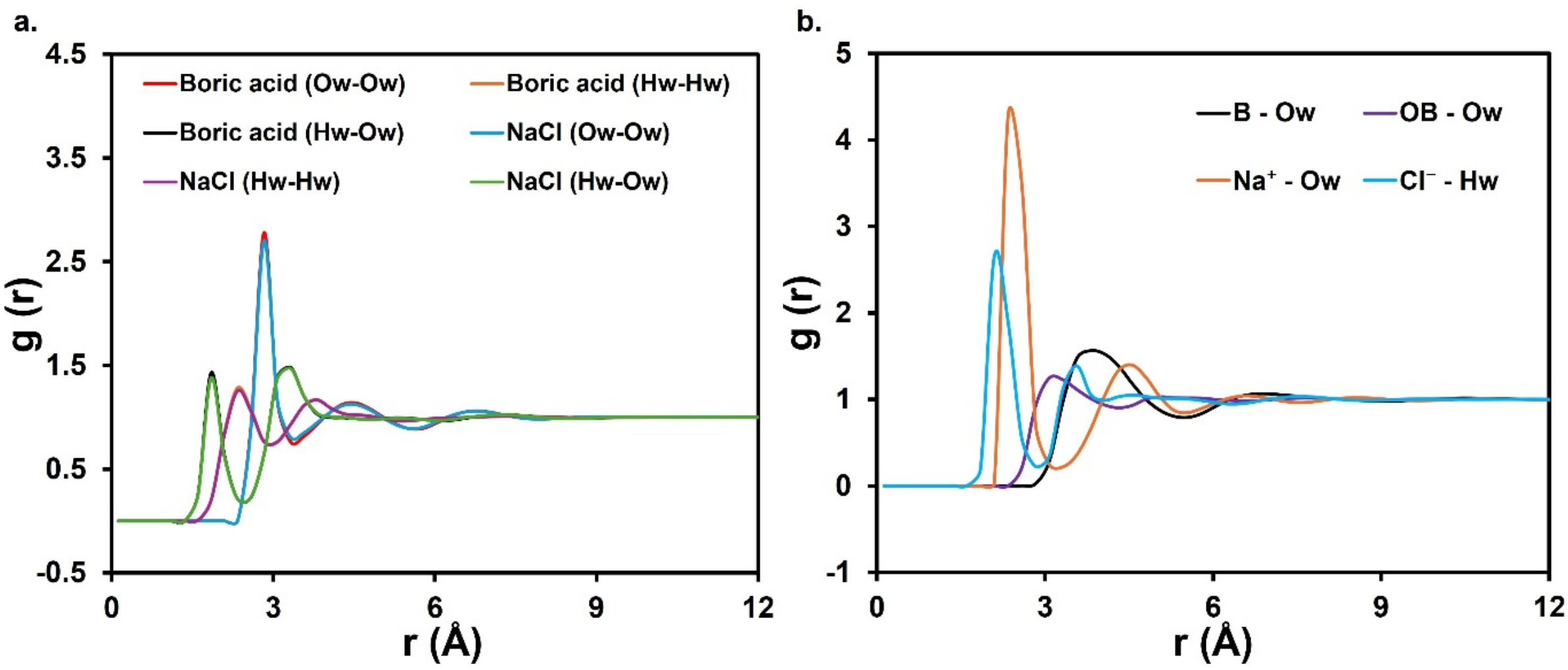

Figure S16. Radial distribution functions (RDFs) for various atomic pairs in NaCl and water + boric acid solution systems. (a) RDFs between water molecule atoms: oxygen-oxygen (Ow-Ow), oxygen-hydrogen (Ow-Hw), and hydrogen-hydrogen (Hw-Hw), comparing NaCl and boric acid solutions. (b) RDFs between solute ions and water: cation/anion-Ow and cation/anion-Hw, illustrating ion-water and boric acid-water interactions.

S10. Lower concentration boric acid simulations

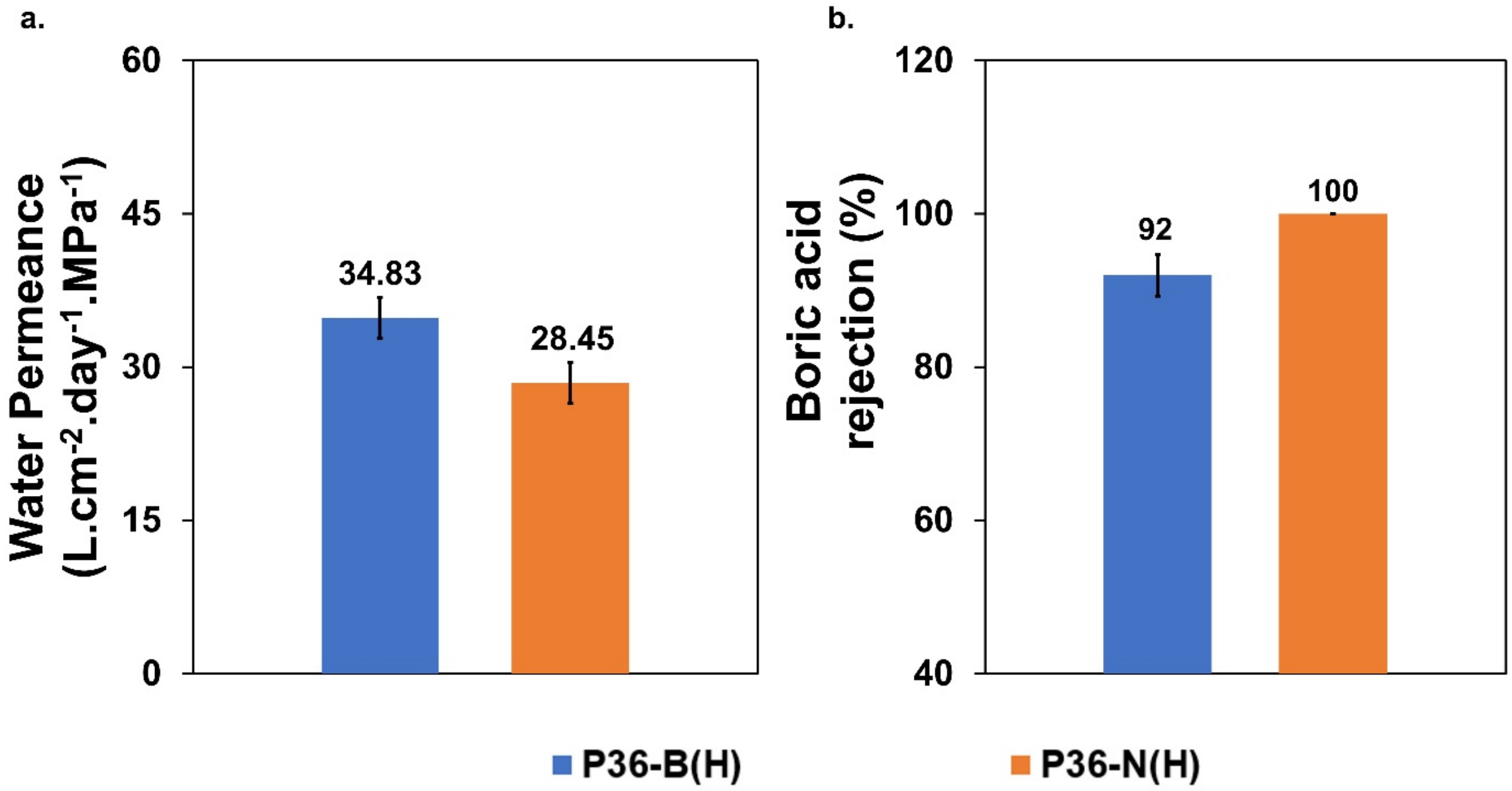

Figure S17. MD simulation results for boric acid separation through P36 nanopores with H-functionalized B- and N-terminated edges. (a) Water permeance per unit membrane area (L cm^{-2} day^{-1} MPa^{-1}) from the feed side to the permeate side for H-functionalized N-terminated and B-terminated nanopores. (b) Boric acid rejection percentage for H-functionalized N-terminated and B-terminated nanopores.

To examine the influence of boric acid concentration on transport behavior, additional simulations were performed at a reduced boric acid concentration (~8.6 g L^{-1}; 5 boric acid molecules in 2000 water molecules) for the P36-B(H) and P36-N(H) pores. Similar transport trends were observed at lower concentration, with the positively charged P36-N(H) pore suppressing boric acid permeation, while the negatively charged P36-B(H) pore allowed boric acid transport.

S11. Details of the density functional theory calculations used to determine the partial charges on the 2D sheets

The hBN unit cell, consisting of 4 atoms (2 boron and 2 nitrogen), was initially optimized using density functional theory (DFT) calculations. The plane wave cutoff energy (E_CUT) and relative cutoff energy (REL_CUT) from cp2k calculations were determined using a 1 meV/atom energy convergence criterion. A 7×7×3 k-point mesh was used for these DFT calculations, with Goedecker-Teter-Hutter pseudopotentials[14] modeling the effect of nuclei and the core electrons. Valence electrons of B, N, C, O, and H were represented using MOLOPT double-zeta short-range basis sets, combined with the PBE exchange-correlation functional[9] and Grimme's D3 dispersion for van der Waals interactions.[15] Next, the hBN unit cell was optimized with E_CUT = 450 Ry, REL_CUT = 60 Ry, and a force criterion of 0.0003 hartree/bohr. The optimized lattice parameters (a = 2.512 Å, b = 2.512 Å, and c = 6.776 Å) were used to construct a 416-atom supercell with a hexagonal arrangement of B and N atoms. Triangular

pores were created by removing 36 atoms, as shown in Figure 1b of the main text, with the edge atoms functionalized with H, OH, and O groups. For graphene, a supercell containing 416 carbon atoms was constructed using the optimized lattice parameters (a = 2.466 Å, b = 2.466 Å, and c = 6.682 Å). A hexagonal nanopore was then created by removing 24 carbon atoms from the center of the sheet, followed by functionalization of the pore edge with H and O atoms, as shown in Figure 1b. Later, geometry optimization for both functionalized and unfunctionalized hBN and graphene sheets were performed using the Broyden-Fletcher-Goldfarb-Shanno algorithm with a force criterion of $0.0003\,\frac{\text{hartree}}{\text{bohr}}$ and a 1×1×1 k-point mesh during supercell optimization.

Partial charges were estimated using the DFT-based DDAP charge algorithm in cp2k, developed by Blöchl.[16] Using the default DDAP parameters in the DENSITY_FITTING section of cp2k, partial charges were assigned to all atoms in the hBN systems. For graphene nanopores, we employed GCUT = 5, NUM_GAUSS = 3, PFACTOR = 1.5, and MIN_RADIUS = 0.264589 for the P24-C(O) pore, whereas we used GCUT = 2.449489, NUM_GAUSS = 4, PFACTOR = 1.5, and MIN_RADIUS = 0.264589 for the P24-C(H) pore, to reproduce near-zero partial charges for carbon atoms located far from the nanopore, consistent with bulk graphene. A cube file was generated with the V_HARTREE_CUBE option to calculate the electrostatic potential from the charge density, allowing for a detailed representation of the potential distribution. To validate these DFT-derived charges, single-point calculations were conducted in LAMMPS with a unit test charge placed perpendicular to the optimized hBN surface, and the electrostatic interaction energy was computed using LAMMPS. This provided a direct comparison of the predicted electrostatic potential from both DFT and MD simulations. Such a comparison is essential, as the DFT potential computed from first-principles calculations acts as the independent theoretical benchmark, since it is the true ab initio potential. As shown in Figure S18, to maintain consistency between DFT and MD potentials, the DFT-predicted partial charges (q_{default}) were rescaled by a factor α, defined as the ratio of the maximum potential value in DFT to that from the partial charges obtained using the default DDAP parameters (before rescaling):

$$\alpha = \frac{|(\text{max potential})_{\text{DFT}}|}{|(\text{max potential})_{\text{partial charges}}|}$$

The rescaled charges were calculated as follows:

$$q_{\text{scaled}} = \alpha q_{\text{default}}$$

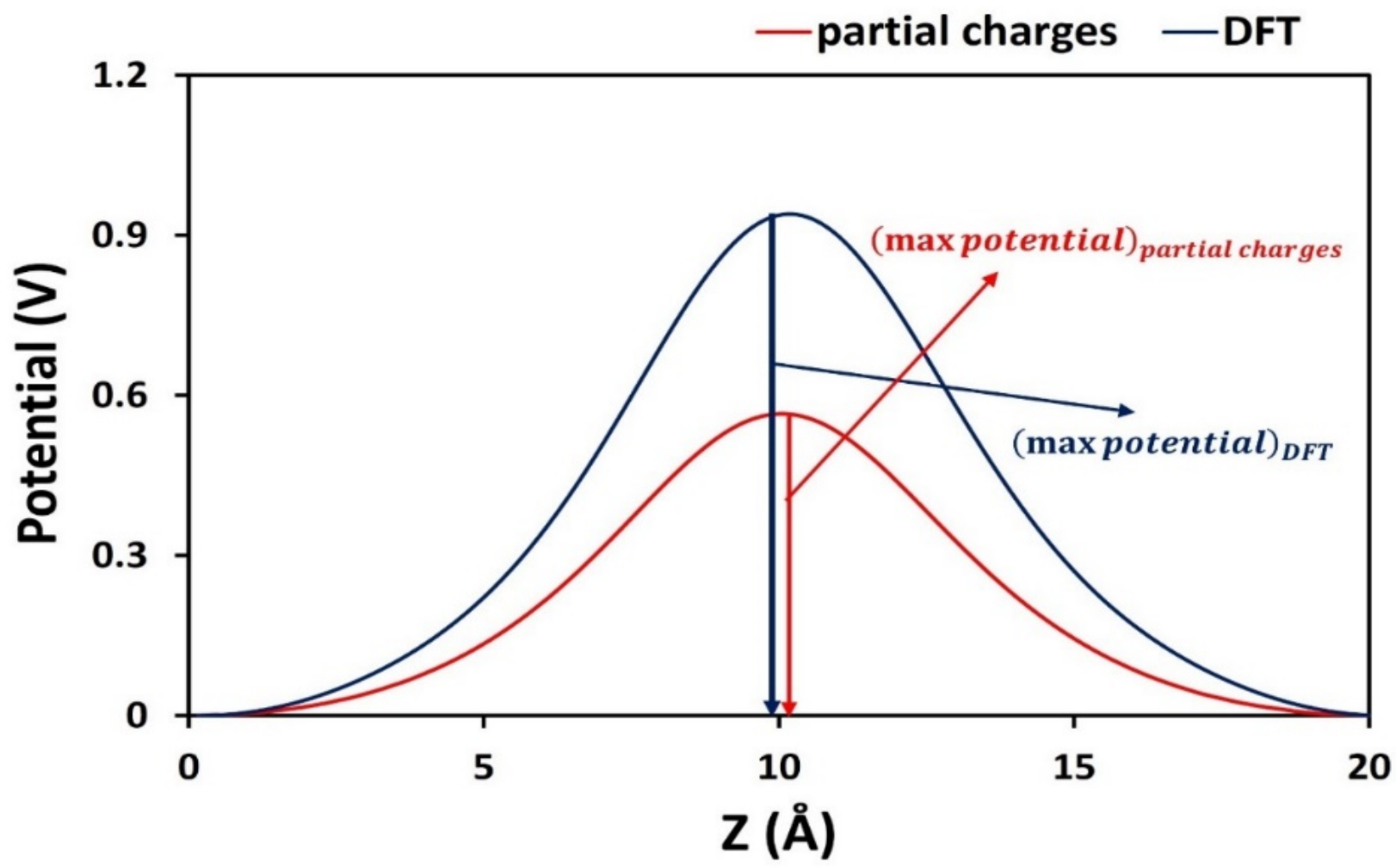

Figure S18. DFT and partial-charge-based potential plots using the default DDAP parameters for the P36-N(H) pore. The maximum potentials from MD partial charges and DFT are labeled. The hBN sheet is positioned at the center of the box at z = 10 Å. The image showing the potential for the P36-N pore is adapted from Ghorai et al.[3] Copyright 2025, American Institute of Physics.

Table S3. Rescaling factor for various pores to generate the rescaled charges.

Pores with default DDAP parameters	Rescaling factor α
**P16-N**	0.87601
**P16-N(H)**	1.68491
**P25-N**	0.82338
**P25-B**	1.44839
**P25-N(H)**	1.72090
**P25-B(H)**	0.52246
**P36-N**	0.81542
**P36-B**	1.38722
**P36-N(H)**	1.71524
**P36-B(H)**	0.51728
**P36-N(3O,9H)**	0.347813
**P36-B(6OH,6H)**	0.519246
**P24-C(O)**	0.6667
**P24-C(H)**	1.0

These rescaled charges were used in molecular dynamics simulations of various hBN pore models to assess water permeation and ion/boron rejection percentage. These simulations allowed for a comprehensive evaluation of the material's performance under realistic conditions.

S12. Performance of P36-B(6OH,6H) nanopore in seawater desalination with different DDAP parameters

Table S4. Water permeance and salt rejection performance with a P36-B(6OH,6H) nanopore.

	**P36-B(6OH,6H) Default DDAP charges**	**P36-B(6OH,6H) Case 1 DDAP charges**
**Water permeance ($L\ cm^{-2}\ MPa^{-1}\ day^{-1}$)**	20.3 ± 2.2	23.8 ± 2.1
**Salt rejection (%)**	99.2 ± 0.7	100 ± 0.0

The DDAP parameters in DENSITY_FITTING used to determine partial charges on all atoms B N O and H include GCUT, which defines the cutoff for charge fitting in reciprocal space, NUM_GAUSS, which specifies the number of Gaussian functions employed to approximate the quantum-mechanical charge distribution at each atomic site, PFACTOR, which regulates the scaling of the Gaussian exponents, and MIN_RADIUS, which indicates the minimum radius of the Gaussian functions utilized in the fitting process.

Table S5. DDAP parameters used in the current study for P36-B(6OH,6H) pore.

**Case**	**GCUT**	**NUM_GAUSS**	**PFACTOR**	**MIN_RADIUS**
**Default**	2.449	3	1.5	0.264589
**Case 1**	5	4	1.5	0.264589

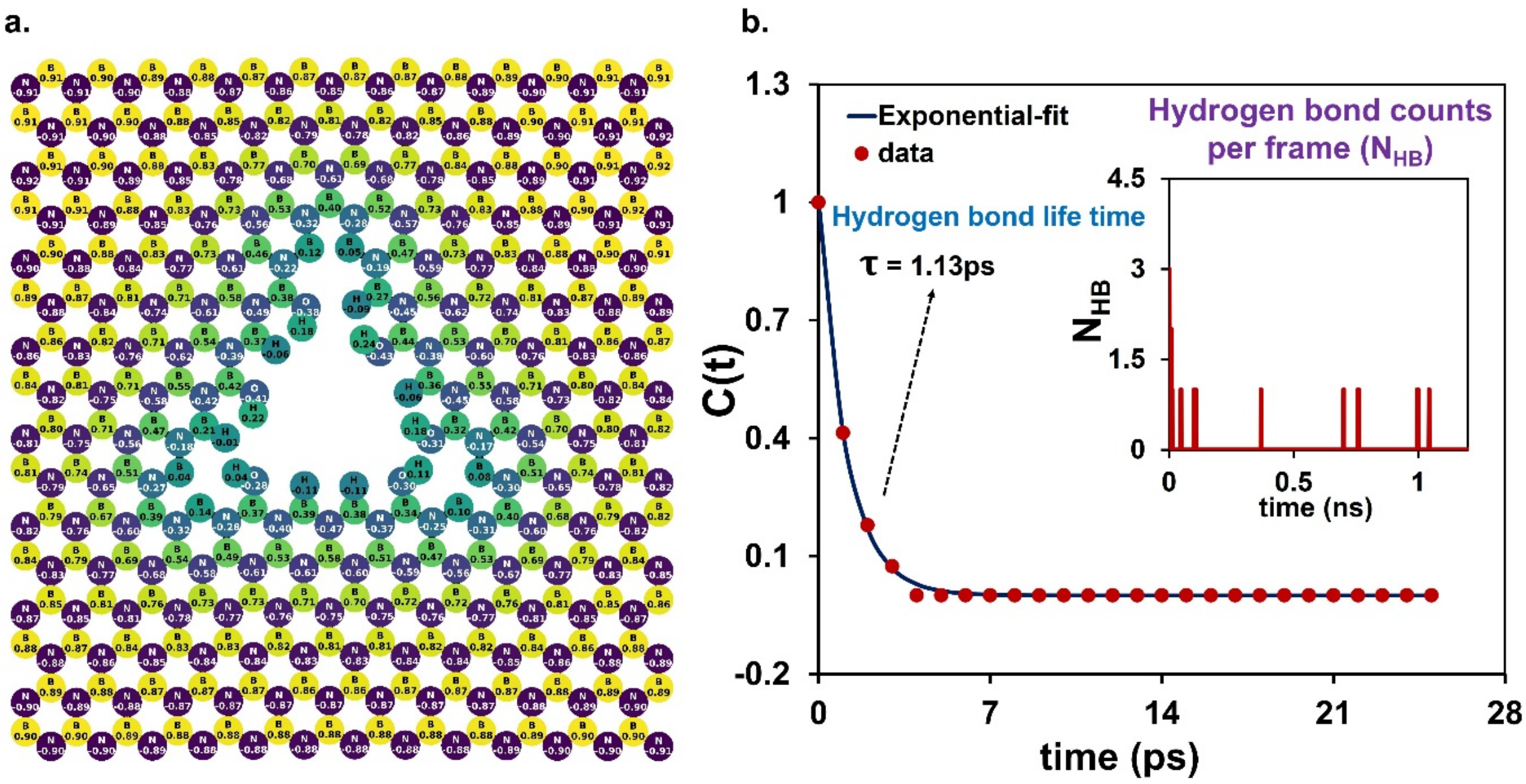

Figure S19. (a) Rescaled partial charge distribution (in elementary charge units, e) computed using the "Case 1" DDAP parameters for the P36-B(6OH,6H) pore. (b) Hydrogen bond analysis of the P36-B(6OH,6H) nanopore, using "Case 1" DDAP charges in MD simulations, demonstrating the hydrogen bond correlation function and hydrogen bond count, with a hydrogen bond lifetime of 1.13 ps. N_{HB} is the number of hydrogen bonds formed during simulation time.

S13. Table of the force-field parameters taken from previous work

Table S6. Force-field parameters of various atoms taken from previous work.

Atom	σ (Å)	$\epsilon \left(\frac{kJ}{mol}\right)$	q (e)	Reference
Na^+ (Salt)	2.21737	1.472356	0.85	Zeron et al.[17]
Cl^- (Salt)	4.69906	0.076923	-0.85	Zeron et al.[17]
C (Piston)	3.4	0.2331456	0.0	Cheng and Steele[18]
B (Membrane)	3.3087	0.2897	Obtained from DFT calculations	Govind Rajan et al.[19]
N (Membrane)	3.2174	0.1979		Govind Rajan et al.[19]
HN (Membrane)	2.42	0.25104		Ghorai et al.[3]
ON (Membrane)	3.12	0.71128		Ghorai et al.[3]
OB (Membrane)	3.12	0.71128		Ghorai et al.[3]
HO (Membrane)	0.0	0.0		Ghorai et al.[3]
HB (Membrane)	2.42	0.29288		Ghorai et al.[3]
H (Water)	0.0	0.0	0.5564	Abascal et al.[20]

O (Water)	3.1589	0.7748768	-1.1128	Abascal et al.[20]

In the molecular dynamics (MD) simulations, we used the TIP4P/2005 water model due to its excellent agreement with experimental values for the diffusion coefficient and water density.[20] Additionally, the LJ parameters recommended by Zeron et al. were adopted for the ions, as these parameters closely replicate the density and viscosity of saltwater solutions across a broad range of concentrations.[17] In Table S6, the partial charges for various atoms and their corresponding Lennard-Jones (LJ) parameters are provided, which are used in the present study. The partial charges for the B and N atoms, as well as the hydrogen atoms bonded to nitrogen (HN), boron (HB), oxygen attached to nitrogen (ON), oxygen in the OH group (OB), and hydrogen in the OH group (HO), were computed using the density derived atomic point (DDAP)[16] charges method through DFT calculations via cp2k.[21] Therefore, these charges are not explicitly stated in this table, but rather can be gleaned from the figures depicted in Section S5.

S14. Impact of applied pressure in the MD simulation outcomes for P36-N(3O,9H) and P36-B(H) nanopores

Table S7. Water permeance and salt rejection performance with P36-N(3O,9H) and P36-B(H) pores under various feed-side pressures.

**P36-N(3O,9H)**	**10 MPa**	**25 MPa**	**50 MPa**	**100 MPa**
**Water permeance ($L\ cm^{-2}\ MPa^{-1}\ day^{-1}$)**	26.0 ± 5.5	30.5 ± 2.8	29.6 ± 0.9	28.3 ± 1.0
**Salt rejection (%)**	100.0 ± 0.0	100.0 ± 0.0	100.0 ± 0.0	99.1 ± 0.9

**P36-B(H)**	**25 MPa**	**50 MPa**	**100 MPa**
**Water permeance ($L\ cm^{-2}\ MPa^{-1}\ day^{-1}$)**	30.2 ± 3.4	29.3 ± 1.6	31.7 ± 0.6
**Salt rejection (%)**	99.2 ± 0.7	99.1 ± 0.9	98.2 ± 1.1

S15. Impact of the number of replicas while averaging the MD simulation outcomes for P36-B(H) and P36-N(3O,9H) nanopores

Table S8. Water permeance and salt rejection performance with P36-B(H) and P36-N(3O,9H) pores.

**P36-B(H)**	**Average (6 replicas)**	**Average (10 replicas)**
**Water permeance ($L\ cm^{-2}\ MPa^{-1}\ day^{-1}$)**	29.3 ± 1.6	29.7 ± 1.1
**Salt rejection (%)**	99.1 ± 0.9	99.3 ± 0.5
**P36-N(3O,9H)**	**Average (6 replicas)**	**Average (10 replicas)**

**Water permeance (L cm^{-2} MPa^{-1} day^{-1})**	29.6 ± 0.9	28.8 ± 0.8
**Salt rejection (%)**	100.0 ± 0.0	100.0 ± 0.0

S16. Convergence analysis of ion rejection across independent simulations

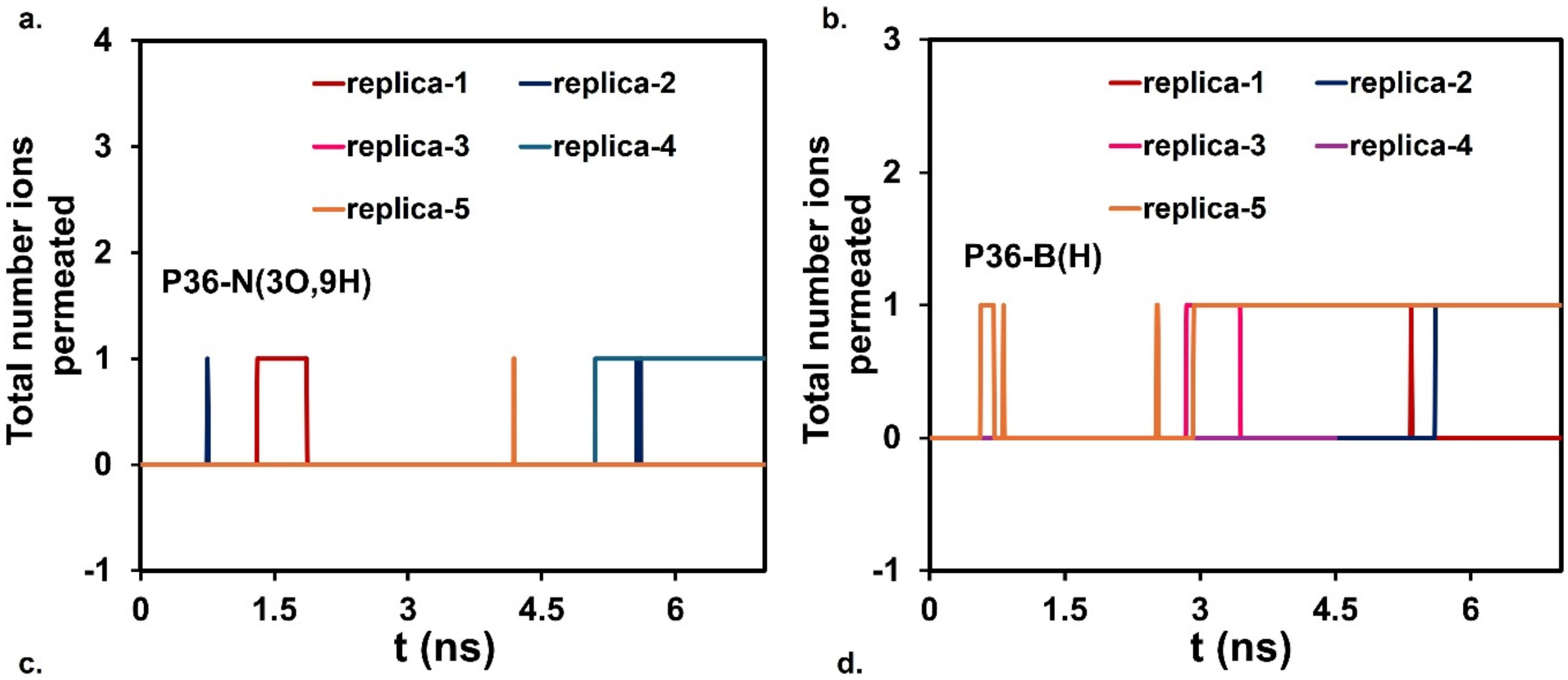

c.

Replica P36-N(3O,9H)	Na^+ crossings	Cl^- crossings	Rejection (%)
1	0	0	100%
2	0	0	100%
3	0	0	100%
4	0	0	100%
5	0	0	100%

d.

Replica P36-B(H)	Na^+ crossings	Cl^- crossings	Rejection (%)
1	0	0	100%
2	0	1	97.72%
3	0	0	100%
4	0	0	100%
5	1	0	97.72%

Figure S20. Convergence analysis of ion rejection for the high-performance hBN nanopores. (a,b) Cumulative ion permeation events as a function of simulation time across six independent simulations for the P36-N(3O,9H) and P36-B(H) pores, respectively. (c,d) Tabulated Na^+/Cl^- crossing statistics together with the corresponding calculated rejection values for each independent replica. The low number of ion crossing events across independent simulations supports the statistical reliability of the reported near-complete rejection behavior.

In Figure S20, the total number of ions is equal to the sum of positive and negative ions in the system. As shown in Figure S20, for the P36-N(3O,9H) pore, ions fluctuate near the pore entrance but return to the feed region during the simulation time, resulting in complete ion rejection within the simulated timescale. In contrast, for the P36-B(H) pore, a small number of ions successfully cross the membrane and migrate toward the permeate side, leading to a slightly lower overall salt rejection (~99.1%) over the given simulation time.

S17. Effect of pore density on water transport and salt rejection

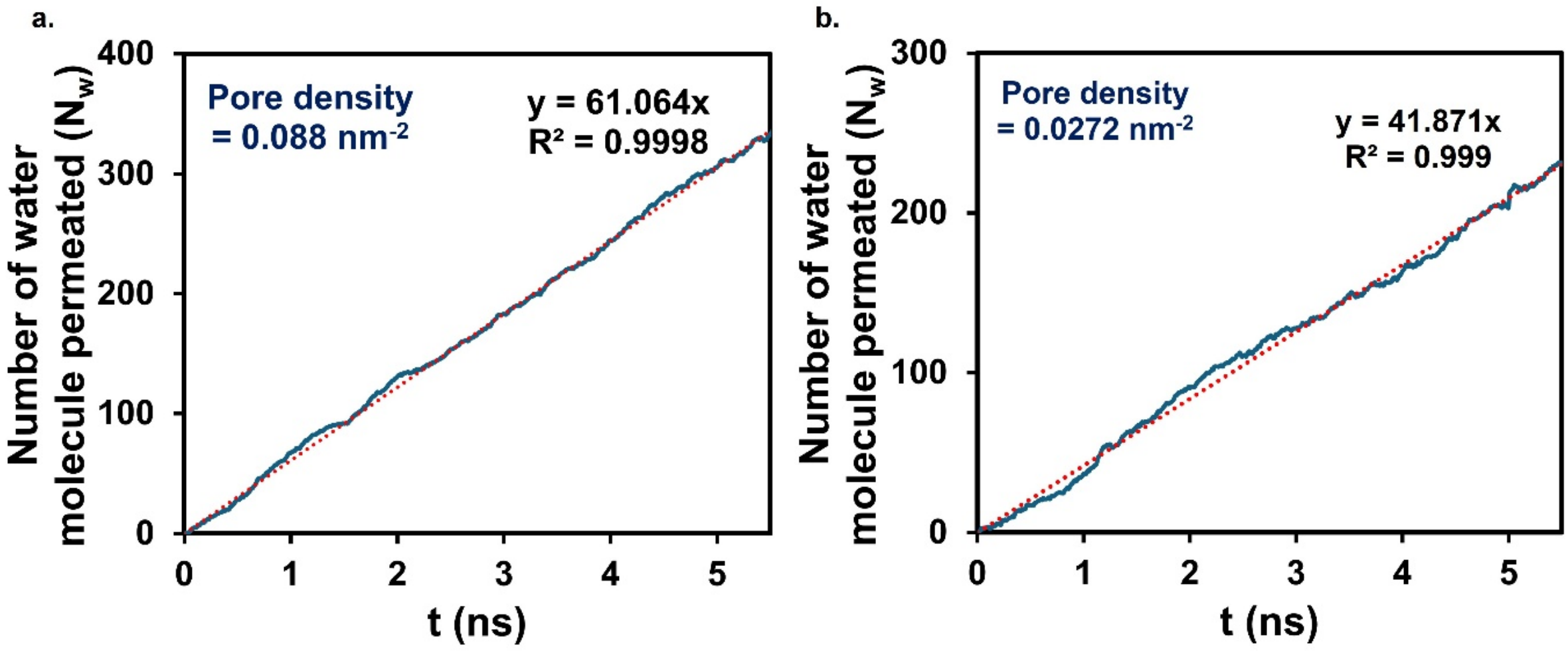

c.

Pore density (nm^{-2}) P36-B(H)	Water flux (N_w/ns)	Salt rejection (%)
0.088	61.06	99.1%
0.0272	41.82	99.8%

Figure S21. Effect of pore density on transport behavior for the representative P36-B(H) hBN nanopore. Number of permeated water molecules as a function of simulation time for membrane models with pore densities of (a) ~0.088 and (b) ~0.0272 nm^{-2}. (c) Corresponding water flux and overall salt rejection values for the two pore densities.

The similar transport behavior observed at different pore densities suggests that the charge-dependent transport characteristics primarily originate from intrinsic pore-level electrostatic interactions. For the lower pore-density (~0.0272 nm^{-2}) simulations, a larger hBN membrane model (~60.2 × 60.1 $Å^2$) was employed, with the feed side containing 8000 water molecules, 88 Na^+ ions, and 88 Cl^- ions, while the permeate side contained 2000 water molecules.

S18. Effect of pore density on water structuring, dipole orientation, and dipole ordering

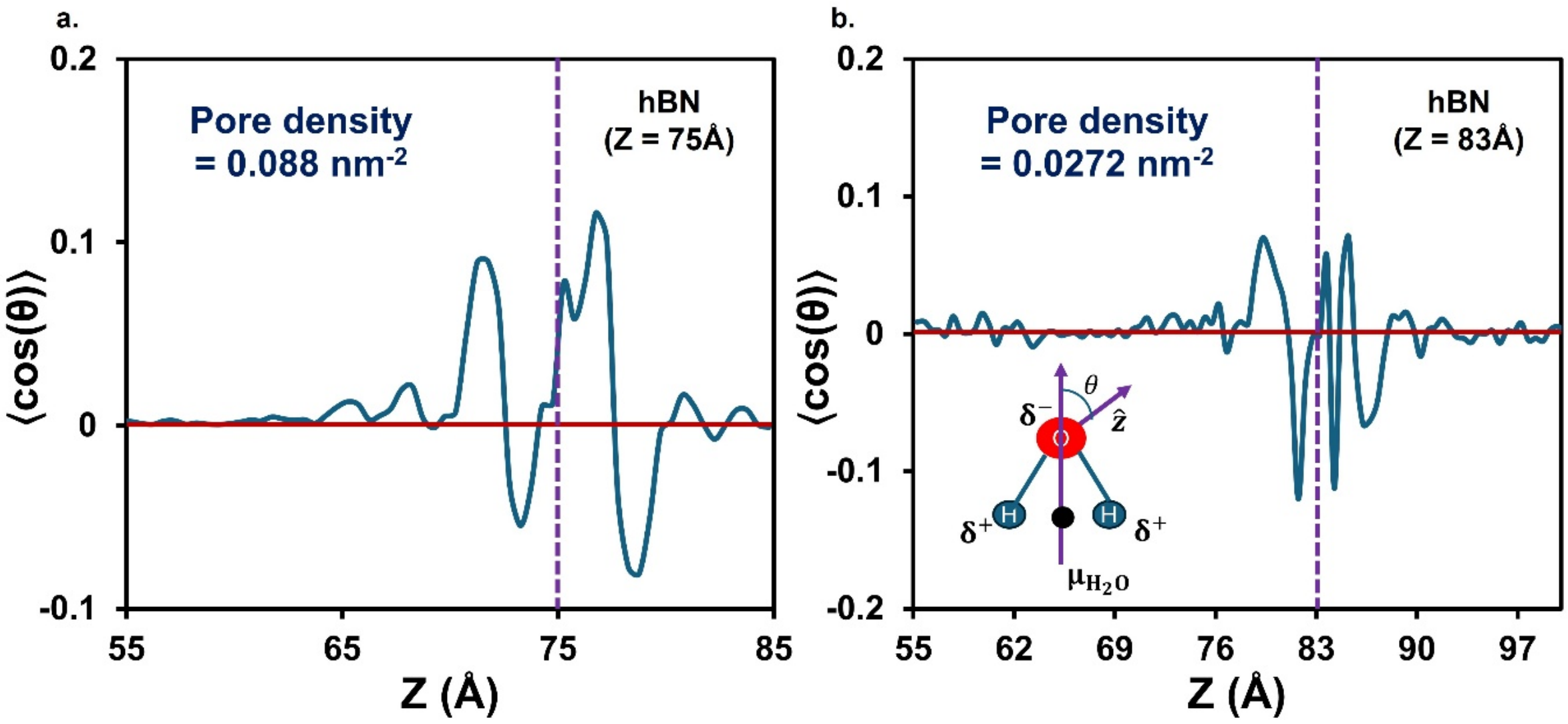

Figure S22. Average water dipole orientation profiles, ⟨cos θ⟩, along the membrane normal (z) direction for the P36-B(H) hBN nanopore at two different pore densities: (a) higher pore density (~0.088 nm^{-2}) and (b) lower pore density (~0.0272 nm^{-2}). As shown here, θ represents the angle between the water molecular dipole vector, defined from the midpoint of the two hydrogen atoms toward the oxygen atom, and the positive z-direction. Values approaching zero away from the nanopore region indicate isotropic bulk-water orientation, whereas oscillatory positive and negative features near the pore region reflect pore-induced orientational ordering of confined water molecules.

To further investigate the effect of pore density on water transport behavior, we analyzed the orientational ordering of water molecules near the nanopore region by calculating the average water dipole orientation profile along the membrane normal (z) direction. The analysis was performed for the representative high-performance P36-B(H) hBN pore at two different pore densities. The water molecular dipole vector was defined using the positions of the oxygen atom and the midpoint between the two hydrogen atoms as $\vec{\mu}_{\mathrm{H_2O}} = \vec{r}_{\mathrm{O}} - \frac{\vec{r}_{\mathrm{H_1}}+\vec{r}_{\mathrm{H_2}}}{2}$, where $\vec{r}_{\mathrm{O}}$, $\vec{r}_{\mathrm{H_1}}$, and $\vec{r}_{\mathrm{H_2}}$ represent the position vectors of the oxygen atom and the two hydrogen atoms of a water molecule, respectively. This definition corresponds to the physical molecular dipole direction pointing from the positive hydrogen side toward the negatively charged oxygen atom. The orientation of each water dipole relative to the membrane normal direction was quantified using $\cos\theta = \hat{\mu}_{\mathrm{H_2O}} \cdot \hat{z}$, where $\hat{\mu}_{H_2O}$ is the normalized (unit) water dipole vector and $\hat{z}$ is the unit vector along the positive z-direction. Here, θ denotes the angle between the water dipole vector and the membrane normal direction. Positive values of $\langle\cos\theta\rangle$ indicate preferential alignment of the oxygen atom toward the positive z-direction, whereas negative values correspond to preferential alignment toward the negative z-direction. The average dipole orientation profile was obtained by spatially binning water molecules along the z-direction and averaging $\cos\theta$ over all water molecules and simulation frames within each bin as $\langle\cos\ \theta\rangle(z) = \frac{1}{N_z}\sum_{\mathrm{i}=1}^{N_z} \cos\theta_i$, where N_z is the number of water molecules within a given z-bin. As shown in Figure S22, water

molecules far from the nanopore region exhibit nearly isotropic orientation with $\langle \cos\theta \rangle \approx 0$, characteristic of bulk liquid water. In contrast, pronounced positive and negative oscillations are observed near the nanopore region, indicating pore-induced orientational ordering of interfacial water molecules arising from local confinement and pore-water interactions. The lower pore-density system (~0.0272 nm^{-2}) exhibits more pronounced oscillatory features with multiple alternating positive and negative regions, including two distinct negative minima, suggesting more structured interfacial water layering near the nanopore. In comparison, the higher pore-density system (~0.088 nm^{-2}) displays a comparatively smoother dipole-orientation profile with more localized orientational variation near the pore region. These differences indicate that pore density can influence collective water structuring and hydrogen-bond organization under periodic boundary conditions, which may contribute to the moderate differences observed in absolute water flux between the two membrane models. Nevertheless, the overall ion rejection trends remain consistent across both pore densities, supporting that the dominant charge-dependent selectivity behavior primarily originates from intrinsic pore-level interactions rather than pore-density effects. The dipole orientation profiles shown in Figure S22 represent averages over multiple independent MD simulations for both the higher and lower pore-density systems.

Table S9. Collective dipole moments (in Debye) of interfacial water molecules near the P36-B(H) nanopore at different pore densities.

**Pore density (nm^{-2})**	$\langle \boldsymbol{M_x} \rangle$	$\langle \boldsymbol{M_y} \rangle$	$\langle \boldsymbol{M_z} \rangle$	$\langle \lvert \boldsymbol{M_z} \rvert \rangle$	$\langle \lvert \boldsymbol{M} \rvert \rangle$
0.088	-0.151	0.0099	-2.121	2.158	18.881
0.0272	-2.274	-0.436	-2.038	3.576	32.591

Table S10. Dipole moment components and total dipole magnitude (in Debye) of the functionalized hBN membrane for different pore densities, calculated from the optimized ground-state structures.

**Pore density (nm^{-2})**	$\boldsymbol{M_x}$	$\boldsymbol{M_y}$	$\boldsymbol{M_z}$	$\lvert \boldsymbol{M} \rvert$
0.088	0.161	297.83	0.0	297.83
0.0272	0.490	1099.447	0.0	1099.448

The collective dipole moments of interfacial water molecules near the nanopore were analyzed using the TIP4P/2005 water model. Water molecules were selected based on the *z*-coordinate of the oxygen atom within a symmetric interfacial region around the membrane center (typically ±3 Å from the membrane plane) to isolate pore-induced water structuring while minimizing bulk-water contributions. For each selected water molecule, the molecular dipole moment vector was calculated as $\vec{\mu}_i = \sum_j q_j \vec{r}_j$, where q_j and $\vec{r}_j$ denote the partial charge and position vector of atom j, respectively. The TIP4P/2005 partial charges used were q_O = -1.1128e for oxygen and q_H = +0.5564e for hydrogen atoms. The collective dipole moment vector of interfacial water was then computed as $\vec{M} = \sum_i \vec{\mu}_i$,

where $\vec{\mu}_i$ is the dipole moment vector of the *i*-th water molecule. The dipole moments were converted from e·Å to Debye using the conversion factor 1 e·Å = 4.803 Debye. Time-averaged values of the Cartesian dipole components ($\langle M_x \rangle$, $\langle M_y \rangle$ and $\langle M_z \rangle$) together with the average magnitude of the membrane-normal dipole component $\langle |M_z| \rangle$ and total collective dipole magnitude $\langle |M| \rangle$ were subsequently computed over the simulation trajectory. Here, $\langle M_z \rangle$ characterizes the average directional polarization of interfacial water along the membrane-normal direction, while $\langle |M_z| \rangle$ and $\langle |M| \rangle$ provide measures of the magnitude of interfacial polarization and overall collective dipolar ordering near the nanopore, respectively. In addition to the dipole orientation profiles, collective polarization analysis of interfacial water molecules was performed using the TIP4P/2005 partial charges. The lower pore-density system exhibited comparatively larger collective dipole magnitudes and more spatially extended interfacial polarization near the nanopore, consistent with the more pronounced oscillatory dipole orientation profiles. These observations suggest enhanced collective water structuring near the pore at lower pore density. In contrast, the higher pore-density system showed higher water flux due to comparatively weaker collective polarization, indicating that differences in interfacial water organization may contribute to the observed variations in absolute water flux. Despite moderate differences in water structuring and water flux, both pore densities exhibited similar ion rejection characteristics.

To further elucidate the origin of the pore-density-dependent water structuring, the dipole moment of the functionalized hBN membrane with different pore densities (P36-B(H)) was calculated from the optimized ground-state structures (Table S10). The lower pore-density membrane (0.0272 nm^{-2}) exhibits a substantially larger total dipole moment (1099.4 Debye) than the higher pore-density membrane (0.088 nm^{-2}, 297.8 Debye), indicating a stronger charge separation within the sheet. A similar trend is evident from the rescaled partial charge distribution shown in Figure S23, where the electrostatic influence of the nanopore extends over a larger area of the hBN membrane at the lower pore density. This enhanced membrane polarization is consistent with the larger collective dipole magnitudes observed for interfacial water molecules in Table S9, where $\langle |M_z| \rangle$ and $\langle |M| \rangle$ increase from 2.16 and 18.88 Debye to 3.58 and 32.59 Debye, respectively, as the pore density decreases. These results suggest that modifications in the membrane charge distribution alter the local electrostatic environment near the nanopore, thereby promoting stronger interfacial water polarization and ordering. The enhanced collective polarization of water at lower pore density provides a plausible explanation for the more pronounced water structuring observed near the pore.

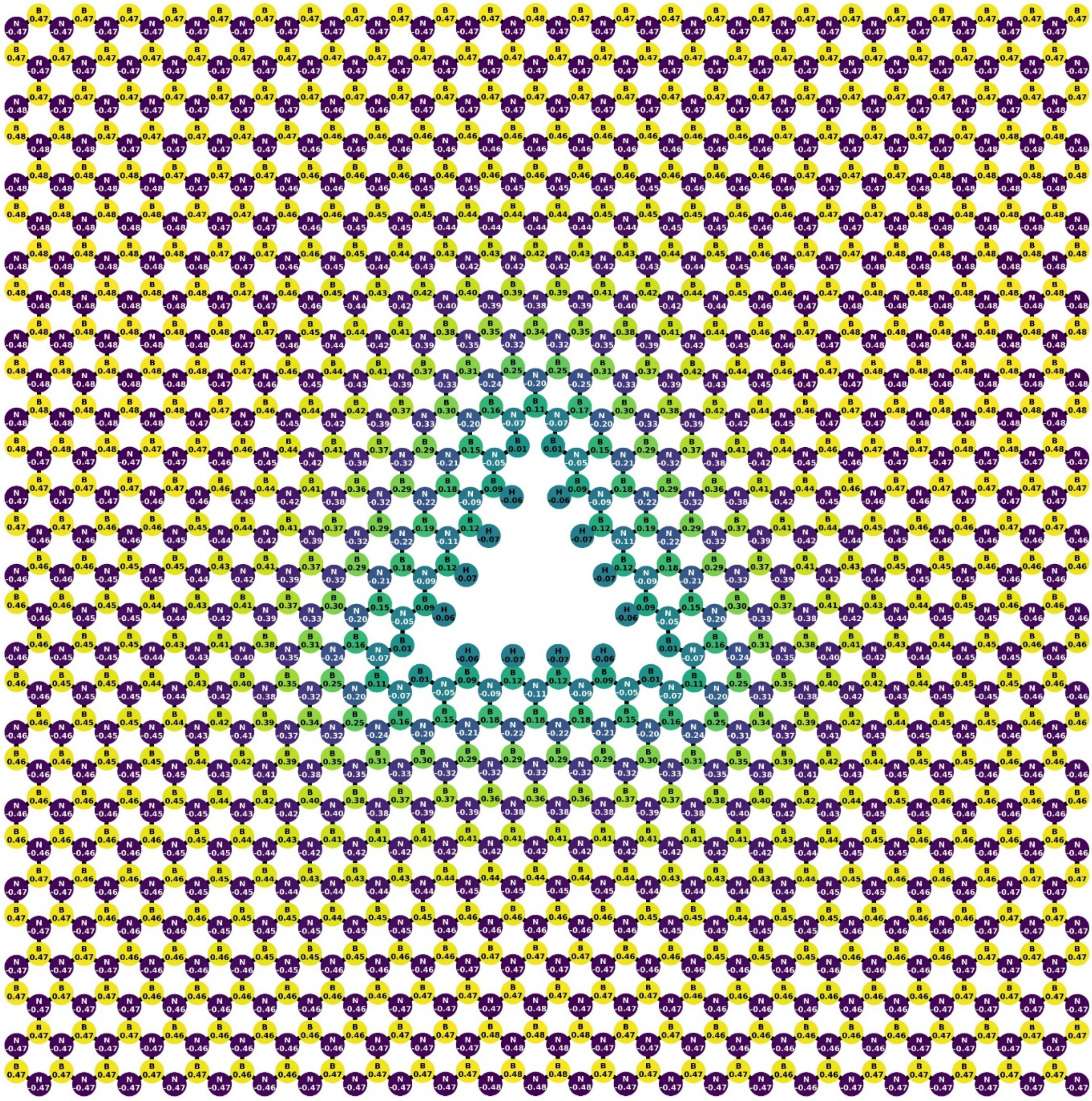

Figure S23. Rescaled partial charge distribution (in elementary charge units, e) computed using the DDAP parameters for P36-B(H) nanopore with a pore density of ~0.0272 nm^{-2}

References

(1) Gao, H.; Shi, Q.; Rao, D.; Zhang, Y.; Su, J.; Liu, Y.; Wang, Y.; Deng, K.; Lu, R. Rational Design and Strain Engineering of Nanoporous Boron Nitride Nanosheet Membranes for Water Desalination. *J. Phys. Chem. C* **2017**, *121* (40), 22105–22113. https://doi.org/10.1021/acs.jpcc.7b06480.

(2) Mantina, M.; Chamberlin, A. C.; Valero, R.; Cramer, C. J.; Truhlar, D. G. Consistent van Der Waals Radii for the Whole Main Group. *J. Phys. Chem. A* **2009**, *113* (19), 5806–5812. https://doi.org/10.1021/jp8111556.

(3) Ghorai, S.; Dhondi, P.; Govind Rajan, A. Molecular Dynamics Simulations of Functionalized hBN Nanopores in Water: *Ab Initio* Force Field and Implications for Water Desalination. *J. Chem. Phys.* **2025**, *162* (4), 044705. https://doi.org/10.1063/5.0242541.

(4) Kresse, G.; Hafner, J. *Ab Initio* Molecular Dynamics for Liquid Metals. *Phys. Rev. B* **1993**, *47* (1), 558–561. https://doi.org/10.1103/PhysRevB.47.558.

(5) Kresse, G.; Furthmüller, J. Efficiency of Ab-Initio Total Energy Calculations for Metals and Semiconductors Using a Plane-Wave Basis Set. *Comput. Mater. Sci.* **1996**, *6* (1), 15–50. https://doi.org/10.1016/0927-0256(96)00008-0.
(6) Kresse, G.; Furthmüller, J. Efficient Iterative Schemes for *Ab Initio* Total-Energy Calculations Using a Plane-Wave Basis Set. *Phys. Rev. B* **1996**, *54* (16), 11169–11186. https://doi.org/10.1103/PhysRevB.54.11169.
(7) Kresse, G.; Joubert, D. From Ultrasoft Pseudopotentials to the Projector Augmented-Wave Method. *Phys. Rev. B* **1999**, *59* (3), 1758–1775. https://doi.org/10.1103/PhysRevB.59.1758.
(8) Blöchl, P. E. Projector Augmented-Wave Method. *Phys. Rev. B* **1994**, *50* (24), 17953–17979. https://doi.org/10.1103/PhysRevB.50.17953.
(9) Perdew, J. P.; Burke, K.; Ernzerhof, M. Generalized Gradient Approximation Made Simple. *Phys. Rev. Lett.* **1996**, *77* (18), 3865–3867. https://doi.org/10.1103/PhysRevLett.77.3865.
(10) Grimme, S.; Ehrlich, S.; Goerigk, L. Effect of the Damping Function in Dispersion Corrected Density Functional Theory. *J. Comput. Chem.* **2011**, *32* (7), 1456–1465. https://doi.org/10.1002/jcc.21759.
(11) Hoover, W. G. Canonical Dynamics: Equilibrium Phase-Space Distributions. *Phys. Rev. A* **1985**, *31* (3), 1695–1697. https://doi.org/10.1103/PhysRevA.31.1695.
(12) Nosé, S. A Unified Formulation of the Constant Temperature Molecular Dynamics Methods. *J. Chem. Phys.* **1984**, *81* (1), 511–519. https://doi.org/10.1063/1.447334.
(13) Dhondi, P.; Govind Rajan, A. Aqueous Chemistry of MoS_2 Nanopores: How Functional Groups Influence Water Permeation and Ion/Boron Rejection, *Nanoscale* **2026**, *18* (17), 9190–9208. https://doi.org/10.1039/D5NR05327G.
(14) Goedecker, S.; Teter, M.; Hutter, J. Separable Dual-Space Gaussian Pseudopotentials. *Phys. Rev. B* **1996**, *54* (3), 1703–1710. https://doi.org/10.1103/PhysRevB.54.1703.
(15) Grimme, S.; Antony, J.; Ehrlich, S.; Krieg, H. A Consistent and Accurate *Ab Initio* Parametrization of Density Functional Dispersion Correction (DFT-D) for the 94 Elements H-Pu. *J. Chem. Phys.* **2010**, *132* (15), 154104. https://doi.org/10.1063/1.3382344.
(16) Blöchl, P. E. Electrostatic Decoupling of Periodic Images of Plane-Wave-Expanded Densities and Derived Atomic Point Charges. *J. Chem. Phys.* **1995**, *103* (17), 7422–7428. https://doi.org/10.1063/1.470314.
(17) Zeron, I. M.; Abascal, J. L. F.; Vega, C. A Force Field of Li^+, Na^+, K^+, Mg^{2+}, Ca^{2+}, Cl^-, and SO_4^{2-} In Aqueous Solution Based on the TIP4P/2005 Water Model and Scaled Charges for the Ions. *J. Chem. Phys.* **2019**, *151* (13), 134504. https://doi.org/10.1063/1.5121392.
(18) Cheng, A.; Steele, W. A. Computer Simulation of Ammonia on Graphite. I. Low Temperature Structure of Monolayer and Bilayer Films. *J. Chem. Phys.* **1990**, *92* (6), 3858–3866. https://doi.org/10.1063/1.458562.
(19) Govind Rajan, A.; Strano, M. S.; Blankschtein, D. Ab Initio Molecular Dynamics and Lattice Dynamics-Based Force Field for Modeling Hexagonal Boron Nitride in Mechanical and Interfacial Applications. *J. Phys. Chem. Lett.* **2018**, *9* (7), 1584–1591. https://doi.org/10.1021/acs.jpclett.7b03443.
(20) Abascal, J. L. F.; Vega, C. A General Purpose Model for the Condensed Phases of Water: TIP4P/2005. *J. Chem. Phys.* **2005**, *123* (23), 234505. https://doi.org/10.1063/1.2121687.
(21) Kühne, T. D.; Iannuzzi, M.; Del Ben, M.; Rybkin, V. V.; Seewald, P.; Stein, F.; Laino, T.; Khaliullin, R. Z.; Schütt, O.; Schiffmann, F.; Golze, D.; Wilhelm, J.; Chulkov, S.; Bani-Hashemian, M. H.; Weber, V.; Borštnik, U.; Taillefumier, M.; Jakobovits, A. S.; Lazzaro, A.; Pabst, H.; Müller, T.; Schade, R.; Guidon, M.; Andermatt, S.; Holmberg, N.; Schenter, G. K.; Hehn, A.; Bussy, A.; Belleflamme, F.; Tabacchi, G.; Glöß, A.; Lass, M.; Bethune, I.; Mundy, C. J.; Plessl, C.; Watkins, M.; VandeVondele, J.; Krack, M.; Hutter, J. CP2K: An Electronic Structure and Molecular Dynamics Software Package - Quickstep: Efficient and Accurate Electronic Structure Calculations. *J. Chem. Phys.* **2020**, *152* (19), 194103. https://doi.org/10.1063/5.0007045.
